# Essential oil composition and biological activities of *Coridothymus capitatus* collected from two locations in Palestine

**DOI:** 10.1371/journal.pone.0357443

**Published:** 2026-09-02

**Authors:** Odey Bsharat, Michel Hanania, Raed Alkowni, Nisreen Al-Hajj, Eman Elsaid, Nawaf Al-Maharik

**Affiliations:** 1 Department of Chemistry, Faculty of Sciences, An-Najah National University, Nablus, Palestine; 2 Department of Chemistry, Faculty of Applied Sciences, Technology and Engineering, Bethlehem University, Bethlehem, Palestine; 3 Department of Biology and Biotechnology, Faculty of Science, An-Najah, National University, Nablus, Palestine; Universidad Autonoma de Chihuahua, MEXICO

## Abstract

*Coridothymus capitatus* (L.) Reichenb. f. (syn. *Thymus capitatus*) is a Mediterranean aromatic herb esteemed for its essential oil rich in bioactive phenolic compounds. In the current study, essential oils (EOs) were obtained by hydrodistillation from fresh and dried leaves of *C. capitatus* collected from Hebron and Bethlehem, Palestine, and evaluated for their chemical composition, antioxidant, antibacterial, and antifungal activities. Gas chromatography-mass spectrometry (GC-MS) analysis showed that oxygenated monoterpenes were the leading class (69.03–74.44%), with thymol and carvacrol as the major constituents. A clear chemotypic divergence was observed between the two locations, with thymol-rich oils in Hebron and carvacrol-rich oils in Bethlehem. Antioxidant activity was evaluated using the DPPH radical-scavenging assay over a concentration range of 0.333–33.33 µg/mL. At the highest experimentally tested concentration (33.33 µg/mL), DPPH inhibition ranged from 28.21 ± 0.76 to 34.40 ± 0.85ᵃ% among the EOs, with Hebron-fresh oil showing the highest activity. Antibacterial tests displayed substantial activity against Gram-positive and Gram-negative bacteria, with MICs between 25 and 100 µg/mL, while antifungal tests revealed strong inhibitory effects against phytopathogenic fungi, particularly *Botrytis californica* and *Fusarium* species, with MICs as low as 6.25 µg/mL. The geographical origin and post-harvest drying influenced the chemical composition and biological activities of the EOs, demonstrating chemotypic variation among Palestinian *C. capitatus* populations. This study provides a comprehensive comparison of EOs from *C. capitatus* populations from two geographically distinct regions of Palestine and evaluates the effects of post-harvest drying on their phytochemical composition and antioxidant, antibacterial, and antifungal activities. These findings contribute to understanding the chemotypic variation of Palestinian *C. capitatus* and highlight its potential as a natural source of bioactive compounds for food preservation, sustainable crop protection, and related applications. *Coridothymus capitatus* (L.) Reichenb. f. (syn. *Thymus capitatus*) is a Mediterranean aromatic herb esteemed for its essential oil rich in bioactive phenolic compounds. In the current study, essential oils (EOs) were obtained by hydrodistillation from fresh and dried leaves of *C. capitatus* collected from Hebron and Bethlehem, Palestine, and evaluated for their chemical composition, antioxidant, antibacterial, and antifungal activities. Gas chromatography-mass spectrometry (GC-MS) analysis showed that oxygenated monoterpenes were the leading class (69.03–74.44%), with thymol and carvacrol as the major constituents. A clear chemotypic divergence was observed between the two locations, with thymol-rich oils in Hebron and carvacrol-rich oils in Bethlehem. Antioxidant activity was evaluated using the DPPH radical-scavenging assay over a concentration range of 0.333–33.33 µg/mL. At the highest experimentally tested concentration (33.33 µg/mL), DPPH inhibition ranged from 37.50 ± 0.83% to 52.08 ± 0.36% among the EOs, with Hebron-fresh oil showing the highest activity. Antibacterial tests displayed substantial activity against Gram-positive and Gram-negative bacteria, with MICs between 25 and 100 µg/mL, while antifungal tests revealed strong inhibitory effects against phytopathogenic fungi, particularly *Botrytis californica* and *Fusarium* species, with MICs as low as 6.25 µg/mL. The geographical origin and post-harvest drying influenced the chemical composition and biological activities of the EOs, demonstrating chemotypic variation among Palestinian *C. capitatus* populations. These findings contribute to understanding the chemotypic variation of Palestinian *C. capitatus* and highlight its potential as a natural source of bioactive compounds for food preservation, sustainable crop protection, and related applications.

## 1. Introduction

Medicinal and aromatic plants are widely acknowledged as rich reservoirs of natural bioactive compounds, particularly essential oils (EOs) that play important roles in food preservation, pharmaceutical formulations, and cosmetic products. Among these plants, members of the Lamiaceae family are especially valued because of their high concentrations of phenolic secondary metabolites and their broad range of biological activities [1–6]. One prominent species within this family is *Coridothymus capitatus* (*Thymbra capitata* (L.) Cav.), commonly referred to as Mediterranean or conehead thyme. This perennial scented bush is native to the Mediterranean basin and has long been used in traditional medicine for the treatment of respiratory, gastrointestinal, and infectious ailments [7,8].

The pharmacological and biological properties of *C. capitatus* are mostly attributed to its volatile oil, often collected from the plant’s aerial parts using hydrodistillation [9–11]. Phytochemical analyses reveal that this EO mostly comprises phenolic monoterpenes, with carvacrol as the major constituent, often accompanied by thymol, *p*-cymene, and γ-terpinene [9–12]. Carvacrol-rich chemotypes are generally recognized as the primary chemical profile of this species, although the qualitative and quantitative composition of the oil may vary according to geographic origin, environmental conditions, and the developmental stage at harvest [7,8].

Significant chemotypic and chemovarietal variability has been documented in *C. capitatus* populations across the Mediterranean region [13–15]. Plants in the Holy Land have unique EO profiles, marked by variations in the relative concentrations of carvacrol and other monoterpenes [16–18]. These differences reflect ecological adaptation and environmental modulation of secondary metabolite production. Comprehensive surveys of aromatic flora from the Holy Land and Sinai have identified multiple chemotypes and chemovarieties of *C. capitatus*, highlighting the marked chemical polymorphism of this species and the importance of geographic origin in determining its biological potential and practical applications [19].

The high concentration of phenolic compounds in the EO of *C. capitatus* is directly associated with its remarkable antioxidant properties. Multiple investigations have confirmed that carvacrol and thymol possess strong radical-scavenging and lipid peroxidation-inhibitory activities, as demonstrated by established *in vitro* assays such as DPPH and ABTS [1–6]. These effects are primarily attributed to their phenolic hydroxyl groups, which enable hydrogen atom or electron donation to neutralize reactive oxygen species and reduce oxidative stress [20]. In addition to its antioxidant potential, EO of *C. capitatus* exhibits antibacterial activity against both Gram-positive and Gram-negative bacteria, as well as antifungal activity against several pathogenic fungi [3,7,11]. These antimicrobial effects are largely attributed to phenolic monoterpenes, particularly carvacrol and thymol, which disrupt microbial cell membranes, increase membrane permeability, and induce leakage of intracellular constituents, ultimately resulting in cell death [21]. Comparable membrane-disruptive antibacterial and antifungal activities of phenolic EO constituents have been demonstrated in oregano- and thyme-based systems [22]. The medicinal relevance of this species is further supported by reports describing anti-inflammatory, cytotoxic, and enzyme-inhibitory activities [19–27].

Despite several studies investigating the chemical composition and biological properties of *C. capitatus* EO in Mediterranean countries, substantial knowledge gaps persist [9,10,12–15,17,19]. Previous researches have largely focused on populations from individual terrestrial regions or specific biological activities, making direct comparisons difficult because of differences in sampling locations, plant materials, phenological stages, extraction methods, and biological assays. Moreover, information on Palestinian populations remains rare, despite Palestine representing a unique biogeographical region with substantial disparity in altitude, climate, precipitation, and soil characteristics over relatively short distances. Such environmental diversity is expected to affect the biosynthesis of secondary metabolites and promote the development of distinct chemotypes. However, no earlier study has systematically compared the chemical composition and biological activities of EOs from fresh and dried leaves collected from geographically distinct Palestinian populations under identical experimental conditions. Consequently, the combined effects of geographical origin and post-harvest drying on EO composition and biological activity remain poorly understood.

We hypothesized that the distinct environmental conditions of the Hebron and Bethlehem regions, together with post-harvest drying, influence the biosynthesis of volatile secondary metabolites, resulting in distinct EO chemotypes and measurable differences in antioxidant and antimicrobial activities.

Accordingly, this study categorized the chemical composition of EOs from fresh and dried *C. capitatus* leaves collected from the Hebron and Bethlehem regions of Palestine and assessed their antioxidant, antibacterial, and antifungal activities under standardized experimental conditions. By combining phytochemical characterization with biological evaluation, this work provides the first direct comparison of geographically distinct Palestinian populations before and after drying, offering new understandings into the effects of environmental variation and post-harvest processing on *C. capitatus* chemotypes.

## 2. Materials and methods

### 2.1. Species collection and identification

*C. capitatus* leaves were gathered from southern Hebron and northern Bethlehem, Palestine, in early May 2025. Although moisture content was not quantitatively determined, all plant materials were collected at the same phenological stage and under similar harvesting conditions. The plant was identified, and voucher specimens were deposited at the An-Najah National University Herbarium under voucher numbers Pharm-PCT-2929a and b. Following collection, the leaves from each location were divided into two portions. One portion was used immediately as fresh material for EO extraction, while the second portion was shade-dried at ambient temperature (25 ± 3 °C) and relative humidity (55 ± 4%), and then stored at 4 °C until extraction. Moisture content was not directly quantified; therefore, comparisons between fresh and dried material are reported on a fresh-weight basis and should be interpreted with caution.

### 2.2. Extraction of essential oil

The harvested *C. capitatus* leaves were mechanically shredded into small fragments to improve the extraction of the EOs and subsequently divided into two parts for recurrent extraction. Fifty grams of shredded leaves underwent hydro-distillation using a Clevenger apparatus for three hours. The resulting oil was extracted with dichloromethane (DCM, 50 mL x 2), and the combined organic layers were dehydrated using Na₂SO₄. Consequently, the DCM was evaporated under reduced pressure, yielding 1.60 ± 0.10% and 1.23 ± 0.15% of pale-yellow oil from the fresh leaves of *C. capitatus* from Hebron and Bethlehem, respectively. In contrast, the dried leaves of *C. capitatus* from Hebron and Bethlehem produced 2. 2.23 ± 0.15% and 1.30 ± 0.10%, respectively. Because the moisture content of the fresh plant material was not determined, yield comparisons between fresh and dried samples are influenced by inherent differences in water content and should not be interpreted as absolute extraction efficiency differences. The oils were contained in a non-transparent vessel and maintained in a refrigerator at 4 °C until needed. Each sample type was extracted independently in triplicate, and the reported EO yields and chemical compositions represent the mean values obtained from three independent extractions.

### 2.3. Qualitative and quantitative analysis of the extracted EOs

The phytochemical constituents of *C. capitatus* EOs were identified qualitatively and quantitatively by gas chromatography coupled with a PerkinElmer Clarus 560 mass spectrometer. Separation was performed on a PerkinElmer Elite-5 fused-silica capillary column (30 m × 0.25 mm i.d., 0.25 µm film thickness). The oven temperature was programmed from 50°C (held for 5 min) to 280°C at a rate of 4°C min ⁻ ¹. Helium was used as the carrier gas at a constant flow rate of 1.0 mL min ⁻ ¹. A 0.2 µL aliquot of the EO diluted in acetonitrile was injected in split mode (split ratio 1:50), with the injector maintained at 250°C.

The EO constituents were identified by comparison of their mass spectra and retention indices with those of 15 authentic reference compounds analyzed under identical chromatographic conditions. Additional compounds were tentatively identified by comparison of their mass spectra with the NIST/EPA/NIH Mass Spectral Library (NIST 17) and by manual verification using the NIST Chemistry WebBook (SRD 69). Compound assignments were further confirmed by comparing their experimentally determined Kovats (linear) retention indices (RIs), calculated using a homologous series of n-alkanes (C_6_–C_30_) analyzed under identical chromatographic conditions, with published literature values obtained on the same stationary phase.

### 2.4. DPPH Free radical scavenging assay

The antioxidant activity of the EOs was evaluated using the DPPH free-radical-scavenging assay at concentrations ranging from 0.333 to 33.33 µg/ml [28–32]. The percentage of DPPH radical inhibition was calculated from the absorbance of the DPPH control and sample according to the following equation:


$$\begin{array}{c} \%𝐢𝐧𝐡𝐢𝐛𝐢𝐭𝐢𝐨𝐧=[(𝐀\_𝐜𝐨𝐧𝐭𝐫𝐨𝐥-𝐀\_𝐬𝐚𝐦𝐩𝐥𝐞)/𝐀\_𝐜𝐨𝐧𝐭𝐫𝐨𝐥]\times 100 \end{array}$$

Each concentration was analyzed in triplicate, and the results were expressed as mean ± SD. Because 50% inhibition was not consistently achieved by all EOs samples within the experimentally tested concentration range, IC₅₀ values were not determined or extrapolated. Instead, the antioxidant activity of the EOs was compared based on the percentage of DPPH radical inhibition at the highest experimentally tested concentration (33.33 µg/mL).

### 2.5. Determination of minimum inhibitory concentration (MIC)

The minimum inhibitory concentrations (MICs) of the EOs against bacterial strains were determined using a broth microdilution procedure based on the CLSI M07 guideline [33]. Antifungal MICs were determined using a broth microdilution procedure adapted from the CLSI M38 guideline [34]. Because CLSI M38 was developed primarily for clinically relevant filamentous fungi, the procedure was modified according to the growth characteristics of the phytopathogenic fungi investigated, particularly with respect to incubation temperature and duration.

The antimicrobial activity was evaluated against eight phytopathogenic fungi, *Alternaria alstroemeriae*, *Botrytis californica*, *Fusarium foetens*, *Penicillium expansum*, *Fusarium equiseti*, *Fusarium fujikuroi*, *Macrophomina tecta*, and *Paecilomyces niveus*, and four human-pathogenic bacterial strains: *Bacillus cereus*, *Staphylococcus aureus*, *Escherichia coli*, and *Klebsiella pneumoniae*.

The fungal isolates were cultured on potato dextrose agar (PDA) at 25–28 °C for 5–7 days. Spore suspensions were prepared in sterile saline or sterile distilled water and adjusted to approximately 1 × 103–5 × 103 CFU/mL. Bacterial suspensions were adjusted to the turbidity of a 0.5 McFarland standard, corresponding to approximately 1.5 × 108 CFU/mL, and subsequently diluted to the inoculum concentration required for the assay.

EO stock solutions were prepared at a concentration of 200 µg/mL in Mueller–Hinton broth containing 20% (v/v) DMSO. Two-fold serial dilutions were prepared using sterile Mueller–Hinton broth for the antibacterial assays and RPMI 1640 medium for the antifungal assays, producing final EO concentrations of 100, 50, 25, 12.5, 6.25, 3.125, 1.563, 0.781, 0.391, and 0.195 µg/mL. All dilutions were prepared aseptically in sterile 96-well microtiter plates. Corresponding solvent controls containing equivalent DMSO concentrations without EO were included to determine whether the solvent affected microbial growth. The test organisms were inoculated into the wells containing the serially diluted EOs and into the growth-control wells. The growth control contained the appropriate assay medium and microbial inoculum without EO, whereas the sterility control contained uninoculated assay medium without EO. The sterility control was used to confirm the absence of microbial contamination. Doxycycline was tested separately as the positive reference control for the antibacterial assays. Commercial phenol (90%) and commercial bleach containing 3% available sodium hypochlorite were tested separately as practical disinfection reference controls for the antifungal assays. These substances were not regarded as conventional antifungal agents or agricultural fungicides. Therefore, their MIC values were reported only as reference values under the conditions of the present assay and were not used to claim comparative superiority of the EOs. Following inoculation, the fungal plates were incubated at 25–28 °C for 3–5 days, depending on the growth characteristics of each isolate, whereas the bacterial plates were incubated at 35 °C for 16–20 h. The MIC was defined as the lowest EO concentration that completely inhibited visible microbial growth at the end of the incubation period. Bacterial growth was assessed visually, whereas fungal growth was evaluated visually with the aid of a dissecting microscope. Each concentration was tested in three technical replicate wells, and the complete experiment was independently repeated three times.

### 2.6. Statistical Analysis

Results are presented as mean ± standard deviation (SD) of three replicate measurements (*n* = 3). Differences in DPPH inhibition among the four *C. capitatus* EO samples at 33.33 µg/mL were analysed using one-way analysis of variance (ANOVA), followed by Tukey’s multiple-comparison post hoc test. Differences were considered statistically significant at *p* < 0.05. The corresponding ANOVA and Tukey post hoc results are provided in S1 Table S5 in S1 File.

## 3. Results and discussion

### 3.1. Chemical Composition

The chemical compositions of EOs derived from fresh and dried leaves of *C. capitatus*, sourced from Hebron and Bethlehem, are presented in Table 1. A total of 27 compounds were identified in EOs from fresh leaves and 25 compounds from dried leaves gathered in Hebron, while 22 and 20 compounds were recognized in EOs from fresh and dried leaves found in Bethlehem, respectively. Several additional chromatographic peaks were detected at trace levels (<0.1% each). As these peaks could not be confidently identified using authentic reference standards, NIST 17 mass spectral library matching, and retention index comparison, they were excluded from Table 1 because of their negligible contribution to the overall EO composition. The findings indicate that both geographic location and post-harvest drying substantially affect the quality profile of *C. capitatus* EOs, as outlined in Table 1. The identified chemicals comprised 99.32–99.68% of the overall EO compositions. Oxygenated monoterpenes were the primary chemical class in all samples (69.03–74.44%), followed by monoterpene hydrocarbons (21.07–25.96%), sesquiterpene hydrocarbons (2.67–4.46%), and minimal quantities of phenylpropanoids (0.02–0.15%). This distribution closely aligns with previous studies indicating that *C. capitatus* EO predominantly comprises oxygenated monoterpenes, especially phenolic derivatives such as thymol and carvacrol [1,3,28].

**Table 1:** Semi-quantitative chemical composition (%) and relative distribution of terpene classes in the EO of *C. capitatus* collected from fresh and dried leaves in Hebron and Bethlehem, Palestine, as determined by GC–MS.

Compound	RI	EOFL-H	EODL-H	EOFL-B	EODL-B
**Monoterpene hydrocarbons**					
Artemisia triene	922	0.70	0.77	1.03	1.17
α-Pinene	928	0.37	0.41	0.61	0.64
Camphene	944	0.18	0.19	0.40	0.26
Myrcene	985	0.98	0.90	1.21	1.35
δ-2-Carene	1000	0.20	0.13	0.19	0.25
α-Terpinene	1011	1.77	1.66	1.80	2.26
*p*-Cymene	1019	6.05	8.15	5.24	7.15
Limonene	1024	0.65	0.62	0.74	0.89
γ-Terpinene	1053	14.43	11.47	9.69	11.72
Terpinolene	1079	0.23	0.22	0.16	0.27
**Oxygenated monoterpenes**					
Terpinen-4-ol	1177	0.58	0.71	1.49	1.29
Thymol methyl ether	1222	0.06	0.19	—	—
Thymol	1292	54.49	54.98	25.20	27.48
Carvacrol	1297	13.10	15.22	46.40	41.05
Linalool	1098	0.65	0.37	1.35	1.14
**Phenylpropanoids**					
Eugenol	1344	0.08	0.03	0.07	0.02
(E)-Methyl cinnamate	1376	0.07	—	—	—
**Identified minor compounds**					
Nepetalactone^a^ (tentative identification)	1354	0.18	0.04	—	—
Cis-Carvyl propanoate	1412	0.11	—	—	—
**Sesquiterpene hydrocarbons**					
(Z)-Caryophyllene	1407	3.15	2.90	3.19	2.49
β-Gurjunene	1425	0.09	0.07	0.08	—
γ-Gurjunene	1477	0.08	0.08	0.06	0.03
Germacrene D	1482	0.59	0.21	0.22	0.06
Cis-Cadina-1,4-diene	1493	0.22	0.14	—	—
γ-Amorphene	1498	0.03	0.01	0.01	—
β-Himachalene	1503	0.06	0.06	0.05	0.02
β-Vetivenene	1565	0.24	0.11	0.15	0.07
**Chemical class ditribution**					
Monoterpene hydrocarbons		25.56	24.52	21.07	25.96
Oxygenated monoterpenes		68.88	71.47	74.44	70.96
Sesquiterpene hydrocarbons		4.46	3.58	3.76	2.67
Phenylpropanoids		0.15	0.03	0.07	0.02
Identified minor compounds		0.29	0.04	0.00	0.00
Total identified (%)		99.34	99.64	99.34	99.61
Unidentified fraction (%)		0.66	0.36	0.66	0.39
Total composition (%)		100.00	100.00	100.00	100.00

RI, retention index on an Elite-5 capillary column; EOFL-H, EO from fresh leaves collected in Hebron; EODL-H, EO from dried leaves collected in Hebron; EOFL-B, EO from fresh leaves collected in Bethlehem; EODL-B, EO from dried leaves collected in Bethlehem.

ᵃ Tentatively identified based on comparison of the experimental retention index (RI) with published mass-spectral and retention-index data; identification was not confirmed using an authentic reference standard.

**Note:** Values represent semi-quantitative relative abundances calculated by TIC peak-area normalization. No FID or compound-specific response factors were applied; therefore, the reported percentages should not be interpreted as absolute concentrations.

Thymol and carvacrol were the primary components of the EOs in all samples, although their relative abundances varied markedly according to geographical origin. In the Hebron samples, thymol was the principal constituent, with relative abundances of 54.49% and 54.98% in the fresh and dried oils, respectively, followed by carvacrol at 13.10% and 15.22%. In contrast, the Bethlehem samples showed a carvacrol-dominant profile, with relative abundances of 41.05–46.40%, whereas thymol represented 25.20–27.48% of the detected constituents. These findings suggest distinct thymol-dominant and carvacrol-dominant compositional profiles among the Palestinian *C. capitatus* oil samples examined in this study, despite the relatively short geographical distance between the sampling locations. Because the GC–MS data were obtained by TIC peak-area normalization without compound-specific response factors, these percentages represent semi-quantitative relative abundances rather than absolute concentrations.

Comparable geographical disparity has been stated across the Mediterranean region [35,36]. The thymol-dominant Hebron population closely resembled wild populations from Northern Cyprus, where thymol was the main component (62.3%), followed by *p*-cymene (10.9%), carvacrol (6.7%), and γ-terpinene (5.1%) [37]. It was also similar to populations from the Greek island of Lemnos, which contained thymol (39.8%), *p*-cymene (31.5%), carvacrol (5.4%), and γ-terpinene (4.2%) [38]. Conversely, the Bethlehem population showed more resemblance to carvacrol-rich chemotypes reported from Morocco and Sicily. In a population gathered from northern Morocco, carvacrol was the principal constituent (79.2%), followed by *o*-cymene (5.1%), α-terpinolene (4.3%), and β-caryophyllene (4.3%) [39]. Correspondingly, flowering populations from northern Sicily contained 76.1–81.2% carvacrol, accompanied by *p*-cymene (5.0–5.8%) and γ-terpinene (2.6–3.8%), whereas thymol was detected only in trace amounts [40,41]. Although the Bethlehem samples contained less carvacrol than the Moroccan and Sicilian populations, their overall composition was consistent with a carvacrol-rich chemotype. Collectively, these comparisons support previous evidence that geographical origin, environmental conditions, plant developmental stage, and genetic variation contribute to the pronounced chemical diversity of *C. capitatus* essential oils throughout the Mediterranean region.

Drying had only a modest effect on the relative distribution of terpene classes. In both locations, drying was associated with a slight increase in oxygenated monoterpenes and a corresponding decrease in monoterpene hydrocarbons. In the Hebron samples, oxygenated monoterpenes increased from 69.03% in fresh leaves to 71.51% in dried leaves, whereas monoterpene hydrocarbons decreased from 25.68% to 24.52%. A similar pattern was observed in the Bethlehem samples. These changes may reflect the preferential loss of more volatile monoterpene hydrocarbons during drying and the resulting relative enrichment of less volatile oxygenated constituents, including thymol and carvacrol [42]. Comparable effects of drying on essential-oil composition have been reported for species of *Clinopodium*, *Origanum*, and *Thymus* [43].

*p*-Cymene (5.24–8.15%) and γ-terpinene (9.69–14.43%) were consistently detected in all samples. These compounds are recognized as biosynthetic precursors of thymol and carvacrol, and their occurrence is therefore consistent with the high abundance of these phenolic monoterpenes [44,45]. Their concentrations were generally within the ranges reported for several Mediterranean populations, although substantially higher levels of these precursors have been observed in some Sicilian, Greek, and Palestinian populations. Such variation may reflect differences in developmental stage, environmental conditions, or the extent of enzymatic conversion of γ-terpinene and *p*-cymene into thymol and carvacrol [46,47]. Sesquiterpene hydrocarbons, including Z-caryophyllene, germacrene D, and β-vetivenene, occurred at relatively low concentrations but may nevertheless contribute to the overall aroma and biological properties of the oils. Phenylpropanoids, including eugenol and E-methyl cinnamate, were detected only in trace amounts, consistent with previous reports indicating that they represent minor constituents of *C. capitatus* EO.

### 3.2. Evaluation of the antioxidant activity

**Fig 1:**
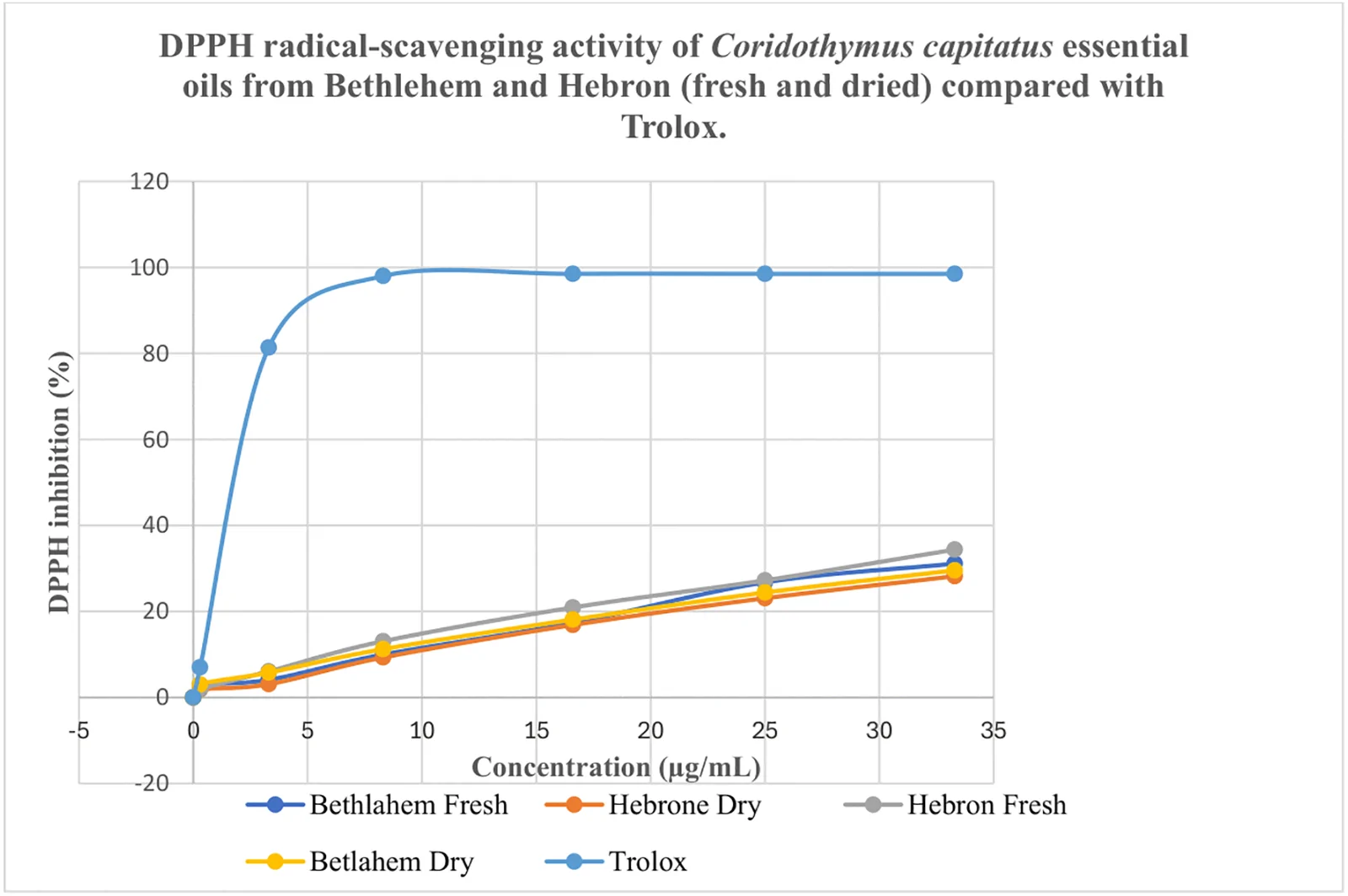
DPPH radical-scavenging activity of *Coridothymus capitatus* EOs from Bethlehem and Hebron (fresh and dried) compared with Trolox.

The antioxidant activity of *C. capitatus* EOs isolated from fresh and dried leaves collected from Hebron and Bethlehem was evaluated using the DPPH free-radical-scavenging assay, with Trolox as a positive control (Fig 1, Table 2). The EOs were tested over a concentration range of 0.333–33.33 µg/mL. At the highest tested concentration (33.33 µg/mL), Hebron-fresh EO exhibited the highest DPPH radical-scavenging activity (34.40 ± 0.85%), followed by Bethlehem-fresh EO (31.15 ± 0.58%), Hebron-dried EO (29.58 ± 1.00%), and Bethlehem-dried EO (28.21 ± 0.76%). One-way ANOVA revealed a significant overall difference among the four EO samples (*p* < 0.001). Tukey’s multiple-comparison test showed that Hebron-fresh EO differed significantly from Bethlehem-fresh EO and both dried EOs. Bethlehem-fresh EO also differed significantly from both dried EOs, whereas no statistically significant difference was **observed** between Hebron-dried and Bethlehem-dried EOs (Table 2). Trolox exhibited substantially greater DPPH radical-scavenging activity than the tested EOs, with an interpolated IC₅₀ value of approximately 2.08 µg/mL.

**Table 2:** DPPH radical-scavenging activity of *Coridothymus capitatus* EOs collected from Hebron and Bethlehem, Palestine, at the highest experimentally tested concentration (33.33 µg/mL).

Sample	DPPH inhibition (%) at 33.33 µg/mL
Hebron fresh	34.40 ± 0.85ᵃ
Bethlehem fresh	31.15 ± 0.58ᵇ
Hebron dry	29.58 ± 1.00ᶜ
Bethlehem dry	28.21 ± 0.76ᶜ

Different superscript letters indicate significant differences according to one-way ANOVA followed by Tukey’s multiple-comparison test (p < 0.05). Values are presented as mean ± SD (n = 3).

The observed variations in antioxidant activity may be related to differences in the chemical composition of the EOs, particularly the relative abundances of phenolic monoterpenes such as thymol and carvacrol, which are recognized contributors to the antioxidant activity of *C. capitatus* and other Lamiaceae EOs [1,20]. The comparatively higher activity of Hebron-fresh EO may be associated with its overall chemical profile. However, antioxidant activity reflects the combined effects of multiple constituents and cannot necessarily be attributed to a single major compound. The phenolic hydroxyl groups of thymol and carvacrol may contribute to radical-scavenging activity through hydrogen donation and free-radical stabilization [47,48].

In the present study, post-harvest drying was associated with decreased DPPH radical-scavenging activity in both geographical populations. The activity of Hebron EO decreased from 34.40 ± 0.85% in the fresh sample to 29.58 ± 1.00% in the dried sample at 33.33 µg/mL. Similarly, the activity of Bethlehem EO decreased from 31.15 ± 0.58% in the fresh sample to 28.21 ± 0.76% in the dried sample. These variations suggest that drying may influence antioxidant activity through changes in the relative abundances or stability of volatile constituents. However, the present results do not establish a direct causal relationship between drying and changes in individual antioxidant compounds. Further chemical analysis would be required to identify the specific constituents responsible for these differences [42].

The antioxidant activity observed in the present study is broadly consistent with previous findings for *C. capitatus* EO. El Ouariachi et al. [16] reported DPPH inhibition of 30 ± 0.7% at 25 µg/mL for an EO obtained from Moroccan *T. capitatus*, increasing to 40 ± 0.3% at 62.5 µg/mL. In the present study, the Palestinian EOs produced 28.21–34.40% inhibition at 33.33 µg/mL. Although the tested concentrations were not identical, these values indicate broadly comparable radical-scavenging activity. Nevertheless, direct quantitative comparison should be made cautiously because of differences in geographical origin, EO composition, plant material, and assay conditions. The Moroccan oil was characterized by carvacrol (13.4%), *p*-cymene (18.9%), geranyl acetate (12.2%), and borneol (10.2%), whereas the Palestinian oils examined here were predominantly thymol- or carvacrol-rich. Other studies from Libya and Tunisia mainly expressed DPPH activity as IC₅₀ values; therefore, their results cannot be directly compared with the percentage inhibition values reported in the present study [9,10,48].

Overall, the present results demonstrate that the DPPH radical-scavenging activity of *C. capitatus* EOs varied according to geographical origin and post-harvest treatment. Among the tested samples, Hebron-fresh EO showed the highest activity at 33.33 µg/mL, whereas both dried EOs showed lower activities and did not differ significantly from each other. These differences may be related to variations in the overall chemical profiles of the oils and the effects of post-harvest processing. Further studies combining antioxidant assays with detailed chemical profiling would help clarify the contributions of individual EO constituents to the observed activity.

### 3.3. Evaluation of the antibacterial activity

The antibacterial efficacy of *C. capitatus* EOs was assessed by the MIC assay against specific Gram-positive (*B. cereus* and *S. aureus*) and Gram-negative (*E. coli* and *K. pneumoniae*) bacteria (Table 3). The findings indicated that all evaluated EOs displayed significant antibacterial efficacy, with MIC values varying from 25 to 100 µg/mL, contingent upon bacterial strain, geographical provenance, and post-harvest processing. Doxycycline, employed as a positive control, showed markedly lower MIC values (0.5–4 µg/mL), consistent with its established antibacterial activity as a tetracycline-class antibiotic that inhibits bacterial protein synthesis through interaction with the 30S ribosomal subunit [47].

**Table 3:** Antibacterial effect MIC (µg/ml) of *Coridothymus capitatus* EO collected from Hebron and Bethlehem in Palestine.

Bacteria	Hebron fresh	Bethlehem fresh	Hebron dry	Bethlehem dry	Doxycycline
**Gram-positive**					
*Bacillus cereus*	50	50	25	25	0.5
*Staphylococcus aureus*	50	100	25	50	0.5
**Gram-negative**					
*Escherichia coli*					
*K. pneumoniae*	100	50	50	50	1

**MIC values represent the final EO concentrations in the assay wells after mixing equal volumes (100 µL) of EO test solution and microbial inoculum. Doxycycline was used as the positive control for the antibacterial assays.**

In general, Gram-positive bacteria have demonstrated a higher degree of susceptibility to EOs than Gram-negative bacteria. In comparison to fresh samples, desiccated samples from Hebron and Bethlehem demonstrated greater efficacy (MIC = 25 µg/mL), with *B. cereus* exhibiting MIC values between 25 and 50 µg/mL. The desiccated EO from Hebron exhibited the highest efficacy (MIC = 25 µg/mL), while the fresh EO from Bethlehem exhibited the lowest efficacy (MIC = 100 µg/mL). A comparable trend was observed for *S. aureus*. The enhanced activity observed during drying can be attributed to a higher relative proportion of oxygenated monoterpenes, particularly thymol and carvacrol, which are recognized for their robust antibacterial properties [49].

Gram-negative bacteria demonstrated elevated MIC values, aligning with their inherent resistance due to an outer barrier abundant in lipopolysaccharides that hinders the transport of hydrophobic substances like EOs [6]. Nonetheless, *K. pneumoniae* exhibited notable sensitivity to the EOs, with MIC values reaching as low as 25 µg/mL for fresh samples from both sites, signifying substantial antibacterial efficacy against this clinically significant pathogen. *E. coli* exhibited moderate susceptibility, with MIC values ranging from 50 to 100 µg/mL.

The antibacterial effectiveness observed in this study is in good agreement with previous reports on *C. capitatus* and related *Thymus* species. Several studies have demonstrated that EOs rich in thymol and carvacrol exhibit strong antibacterial activity against both Gram-positive and Gram-negative bacteria [3,22]. Specifically, *T. capitatus* EO from Mediterranean regions has been reported to inhibit *S. aureus*, *B. cereus*, *E. coli*, and *Klebsiella* species at comparable MIC ranges, with activity strongly correlated to its phenolic monoterpene content [50,51].

Thymol and carvacrol exert their antibacterial effects by compromising bacterial cell membrane integrity, resulting in heightened permeability, leaking of intracellular constituents, and ultimately, cell death [6,21]. The increased activity shown in dried samples, especially against Gram-positive bacteria, may indicate compositional changes after drying that enhance the concentration or relative abundance of these bioactive chemicals, as previously documented for thyme essential oils [52].

The findings affirm that *C. capitatus* EOs from Palestine have extensive antibacterial efficacy, demonstrating significant impacts against Gram-positive bacteria and moderate to strong activity against Gram-negative pathogens. These findings corroborate previous observations about the antimicrobial efficacy of *C. capitatus* and underscore its potential as a natural source of antibacterial compounds for pharmaceutical or food preservation applications.

### 3.4. Antifungal activity of *C. capitatus* EO

The antifungal effectiveness of *C. capitatus* EOs was evaluated against eight phytopathogenic fungi using the MIC assay (Table 4). All assessed EOs showed notable antifungal efficacy, with MIC values ranging from 6.25 to 100 µg/mL, dependent on the fungal species, geographic origin, and post-harvest treatment. Among the assessed fungi, *Botrytis californica* showed the greatest susceptibility, revealing the lowest MIC values (6.25–50 µg/mL), particularly for oils extracted from fresh plant leaves. Similarly, *Fusarium foetens* and *Fusarium equiseti* showed notable sensitivity, with MIC values as low as 12.5 µg/mL. In contrast, *Paecilomyces niveus* exhibited reduced sensitivity, since no inhibitory effects were observed at the tested dosages for specific oil samples, indicating species-dependent resistance patterns. The comparatively lower susceptibility of *P. niveus* is biologically relevant because this fungus is a heat-resistant, patulin-producing species known for its persistence in food products and its relative tolerance to environmental and antimicrobial stresses. The lack of complete inhibition by the Hebron fresh and Hebron dry oils may be attributed to differences in their chemical composition, including the intrinsic resistance characteristics of *P. niveus*. These findings emphasize that antifungal activity varies among EO chemotypes.

**Table 4:** Antifungal effect MIC (µg/ml) of *Coridothymus capitatus* EO collected from Hebron and Bethlehem in Palestine.

Phytopathogenic fungus	Hebron fresh	Bethlehem fresh	Hebron dry	Bethlehem dry	Phenol*	Bleach*
*Alternaria alstroemeria*	50	50	100	100	3516	1875
*Fusarium foetens*	12.5	25	12.5	50	3516	3750
*Penicillium expansum*	50	50	50	50	3516	1875
*Botrytis californica*	6.25	12.5	25	50	3516	1875
*Fusarium equiseti*	25	25	25	25	3516	1875
*Fusarium fujikuroi*	50	25	50	25	3516	1875
*Macrophomina tecta*	50	25	50	25	3516	1875
*Paecilomyces niveus*	>100	25	>100	25	3516	1875

*MIC values represent the final EO concentrations in the assay wells after mixing equal volumes (100 µL) of EO test solution and fungal inoculum. Phenol (90%) and 3% sodium hypochlorite were included as reference disinfection controls rather than conventional antifungal agents or agricultural fungicides; therefore, their MIC values were reported for reference purposes only and were not used for direct superiority comparisons with the essential oils. > 100 indicates that complete inhibition was not observed at the highest final EO concentration tested (100 µg/mL).

The antifungal activity identified in this study aligns with prior investigations on *C. capitatus* and related Thymus species, wherein EOs abundant in phenolic monoterpenes demonstrated considerable inhibitory effects against phytopathogenic fungi, including *Alternaria*, *Fusarium*, *Penicillium*, and *Botrytis* spp. [10,11,53]. The EO of *T. capitatus* from the editerranean region has demonstrated full inhibition of *Penicillium digitatum* and *Botrytis cinerea* at low doses (250 ppm), indicating its potential as a natural antifungal agent [11].

The high levels of oxygenated monoterpenes, particularly thymol and carvacrol, that predominate the chemical compositions of the examined oils are the primary reason for the significant antifungal efficacy of *C. capitatus* EO. Phenolic compounds are acknowledged for their antifungal properties, which encompass the disruption of fungal cell membranes, the enhancement of membrane permeability, and the disruption of enzyme systems that are essential for the construction of cell walls and the metabolism of energy [3,22]. Additionally, the antifungal efficacy may be improved by the synergistic interactions between the primary phenolics and minor constituents, including p-cymene and γ-terpinene [21].

The EOs inhibited the tested phytopathogenic fungi with the MIC values ranging from 6.25 to 100 µg/mL, depending on the fungal species and EO sample. The MIC of commercial bleach with 3% available sodium hypochlorite ranged from 1875 to 3750 µg/mL, while the MIC of phenol was 3516 µg/mL under the conditions of the present assay (Table 4). However, an important limitation of the study was the use of phenol and commercial bleach as reference controls. These substances are general disinfectants rather than conventional antifungal agents or agricultural fungicides, which limits the relevance of direct comparisons with the EOs. Accordingly, their MIC values were interpreted only as descriptive reference values under the conditions of the present assay and not as evidence of greater or equivalent antifungal efficacy of the EOs.

The observed antifungal activity of *C. capitatus* EOs may be associated with their high content of phenolic monoterpenes, particularly thymol and carvacrol, which have previously been reported to contribute to antimicrobial activity [3,54]. These findings indicate that *C. capitatus* EOs possess promising in-vitro antifungal activity against several phytopathogenic fungi. Further studies addressing formulation, phytotoxicity, toxicity, stability, and in-vivo or field efficacy are required before practical agricultural applications can be proposed.

## 4. Conclusion

This study provides a systematic assessment of the chemical composition and biological activities of essential oils from *C. capitatus* obtained from fresh and dried plant material collected in Hebron and Bethlehem, Palestine. GC-MS analysis showed that the oils were predominantly composed of oxygenated monoterpenes, particularly thymol and carvacrol, and revealed clear chemotypic differences between the two geographical locations. These findings highlight the influence of geographical origin and post-harvest treatment on the chemical composition of *C. capitatus* essential oils.

The essential oils demonstrated measurable DPPH radical-scavenging activity, with inhibition values at 33.33 µg/mL ranging from 37.50 ± 0.83% to 52.08 ± 0.36%. Hebron-fresh EO exhibited the highest activity, whereas Hebron-dry EO showed the lowest activity. Because 50% inhibition was not consistently achieved across all samples within the experimentally tested concentration range, IC₅₀ values were not determined. The differences in antioxidant activity may be associated with variations in the abundance and relative proportions of phenolic monoterpenes, particularly thymol and carvacrol.

The EOs also exhibited antibacterial activity against both Gram-positive and Gram-negative bacteria, as well as potent antifungal activity against several phytopathogenic fungi, with MIC values as low as 6.25 µg/mL. Post-harvest drying altered both the chemical composition and biological properties of the oils, although its effects differed according to geographical origin and the target microorganism. Collectively, these findings demonstrate that geographical origin, chemotypic composition, and post-harvest processing are important factors influencing the biological properties of *C. capitatus* essential oils. The oils therefore represent promising sources of natural bioactive compounds with potential applications in food preservation and agricultural disease management. Further studies addressing toxicity, formulation, mechanisms of action, and in vivo efficacy are warranted to assess their practical applicability.

## Supporting information

S1 File This file contains Tables S1–S5, including raw DPPH absorbance measurements, recalculated DPPH radical-scavenging activity, summary DPPH inhibition data, DPPH control absorbance values, and one-way ANOVA with Tukey’s multiple-comparison test for DPPH radical-scavenging activity at 33.333 µg/mL. (DOCX)

S1 Fig Graphical abstract. (TIF)
