# Supplementary material for: Essential oil composition and biological activities of Coridothymus capitatus collected from two locations in Palestine
Source: PLoS One. 2026 Sep 2;21(9):e0357443. doi: 10.1371/journal.pone.0357443 (PMC13537545; doi:10.1371/journal.pone.0357443)
Supplement: Supplementary file 1 [file pone.0357443.s001.docx]

**Supplementary Material**

**Supplementary Table S1. Raw absorbance measurements at 517 nm for *Coridothymus capitatus* essential oils and Trolox**

The following tables present the raw absorbance measurements recorded at 517 nm for the DPPH assay. Each concentration was analyzed in triplicate. The concentrations shown represent the final experimental concentrations used in the assay after application of the experimental dilution factor. The control absorbance was used as the reference for calculation of percentage DPPH inhibition.

**A. Bethlehem – Fresh essential oil**

| **Concentration (µg/mL)** | **Replicate 1 (A517)** | **Replicate 2 (A517)** | **Replicate 3 (A517)** |
| --- | --- | --- | --- |
| 0.333 | 0.385 | 0.386 | 0.386 |
| 3.333 | 0.381 | 0.385 | 0.376 |
| 8.333 | 0.349 | 0.362 | 0.361 |
| 16.667 | 0.336 | 0.329 | 0.319 |
| 25.000 | 0.296 | 0.296 | 0.280 |
| 33.333 | 0.272 | 0.272 | 0.276 |

**DPPH control A517 = 0.397**

**B. Bethlehem – Dry essential oil**

| **Concentration (µg/mL)** | **Replicate 1 (A517)** | **Replicate 2 (A517)** | **Replicate 3 (A517)** |
| --- | --- | --- | --- |
| 0.333 | 0.389 | 0.389 | 0.391 |
| 3.333 | 0.383 | 0.383 | 0.388 |
| 8.333 | 0.359 | 0.359 | 0.362 |
| 16.667 | 0.331 | 0.330 | 0.329 |
| 25.000 | 0.304 | 0.307 | 0.305 |
| 33.333 | 0.288 | 0.285 | 0.282 |

**DPPH control A517 = 0.397**

**C. Hebron – Fresh essential oil**

| **Concentration (µg/mL)** | **Replicate 1 (A517)** | **Replicate 2 (A517)** | **Replicate 3 (A517)** |
| --- | --- | --- | --- |
| 0.333 | 0.456 | 0.466 | 0.464 |
| 3.333 | 0.444 | 0.442 | 0.441 |
| 8.333 | 0.409 | 0.413 | 0.406 |
| 16.667 | 0.374 | 0.377 | 0.366 |
| 25.000 | 0.347 | 0.342 | 0.339 |
| 33.333 | 0.309 | 0.313 | 0.305 |

**DPPH control A517 = 0.471**

**D. Hebron – Dry essential oil**

| **Concentration (µg/mL)** | **Replicate 1 (A517)** | **Replicate 2 (A517)** | **Replicate 3 (A517)** |
| --- | --- | --- | --- |
| 0.333 | 0.451 | 0.459 | 0.460 |
| 3.333 | 0.448 | 0.442 | 0.441 |
| 8.333 | 0.418 | 0.418 | 0.418 |
| 16.667 | 0.384 | 0.385 | 0.388 |
| 25.000 | 0.357 | 0.355 | 0.356 |
| 33.333 | 0.328 | 0.337 | 0.330 |

**DPPH control A517 = 0.471**

**E. Trolox**

| **Concentration (µg/mL)** | **Replicate 1 (A517)** | **Replicate 2 (A517)** | **Replicate 3 (A517)** |
| --- | --- | --- | --- |
| 0.333 | 0.415 | 0.412 | 0.417 |
| 1.000 | 0.344 | 0.340 | 0.344 |
| 1.667 | 0.261 | 0.266 | 0.269 |
| 2.333 | 0.191 | 0.195 | 0.203 |
| 3.333 | 0.089 | 0.077 | 0.083 |
| 5.000 | 0.018 | 0.010 | 0.011 |
| 10.000 | 0.006 | 0.007 | 0.007 |

**Trolox/DPPH control A517 = 0.446**

**Supplementary Table S2. Recalculated DPPH radical inhibition (%) for each individual replicate**

Percentage inhibition was calculated for each replicate using the following equation:

**% inhibition = [(A_control − A_sample)/A_control] × 100**

**A. Bethlehem – Fresh essential oil**

| **Concentration (µg/mL)** | **Replicate 1 (%)** | **Replicate 2 (%)** | **Replicate 3 (%)** | **Mean ± SD (%)** |
| --- | --- | --- | --- | --- |
| 0.333 | 3.02 | 2.77 | 2.77 | 2.85 ± 0.15 |
| 3.333 | 4.03 | 3.02 | 5.29 | 4.11 ± 1.14 |
| 8.333 | 12.09 | 8.82 | 9.07 | 9.99 ± 1.79 |
| 16.667 | 15.37 | 17.13 | 19.65 | 17.38 ± 2.16 |
| 25.000 | 25.44 | 25.44 | 29.47 | 26.78 ± 2.33 |
| 33.333 | 31.49 | 31.49 | 30.48 | 31.15 ± 0.58 |

**B. Bethlehem – Dry essential oil**

| **Concentration (µg/mL)** | **Replicate 1 (%)** | **Replicate 2 (%)** | **Replicate 3 (%)** | **Mean ± SD (%)** |
| --- | --- | --- | --- | --- |
| 0.333 | 2.02 | 2.02 | 1.51 | 1.85 ± 0.29 |
| 3.333 | 3.53 | 3.53 | 2.27 | 3.11 ± 0.73 |
| 8.333 | 9.57 | 9.57 | 8.82 | 9.32 ± 0.44 |
| 16.667 | 16.62 | 16.88 | 17.13 | 16.88 ± 0.26 |
| 25.000 | 23.43 | 22.67 | 23.17 | 23.09 ± 0.39 |
| 33.333 | 27.46 | 28.21 | 28.97 | 28.21 ± 0.76 |

**C. Hebron – Fresh essential oil**

| **Concentration (µg/mL)** | **Replicate 1 (%)** | **Replicate 2 (%)** | **Replicate 3 (%)** | **Mean ± SD (%)** |
| --- | --- | --- | --- | --- |
| 0.333 | 3.18 | 1.06 | 1.49 | 1.91 ± 1.12 |
| 3.333 | 5.73 | 6.16 | 6.37 | 6.09 ± 0.33 |
| 8.333 | 13.16 | 12.31 | 13.80 | 13.09 ± 0.75 |
| 16.667 | 20.59 | 19.96 | 22.29 | 20.95 ± 1.21 |
| 25.000 | 26.33 | 27.39 | 28.03 | 27.25 ± 0.86 |
| 33.333 | 34.39 | 33.55 | 35.25 | 34.40 ± 0.85 |

**D. Hebron – Dry essential oil**

| **Concentration (µg/mL)** | **Replicate 1 (%)** | **Replicate 2 (%)** | **Replicate 3 (%)** | **Mean ± SD (%)** |
| --- | --- | --- | --- | --- |
| 0.333 | 4.25 | 2.55 | 2.34 | 3.05 ± 1.03 |
| 3.333 | 4.88 | 6.16 | 6.37 | 5.80 ± 0.80 |
| 8.333 | 11.25 | 11.25 | 11.25 | 11.25 ± 0.00 |
| 16.667 | 18.47 | 18.26 | 17.62 | 18.12 ± 0.44 |
| 25.000 | 24.20 | 24.63 | 24.42 | 24.42 ± 0.22 |
| 33.333 | 30.36 | 28.45 | 29.94 | 29.58 ± 1.00 |

**E. Trolox**

| **Concentration (µg/mL)** | **Replicate 1 (%)** | **Replicate 2 (%)** | **Replicate 3 (%)** | **Mean ± SD (%)** |
| --- | --- | --- | --- | --- |
| 0.333 | 6.95 | 7.62 | 6.50 | 7.02 ± 0.56 |
| 1.000 | 22.87 | 23.77 | 22.87 | 23.17 ± 0.52 |
| 1.667 | 41.48 | 40.36 | 39.69 | 40.51 ± 0.91 |
| 2.333 | 57.17 | 56.28 | 54.48 | 55.98 ± 1.35 |
| 3.333 | 80.04 | 82.74 | 81.39 | 81.39 ± 1.35 |
| 5.000 | 95.96 | 97.76 | 97.53 | 97.08 ± 0.98 |
| 10.000 | 98.65 | 98.43 | 98.43 | 98.51 ± 0.13 |

**Supplementary Table S3. Summary of recalculated DPPH radical-scavenging activity**

| **Sample** | **Concentration (µg/mL)** | **Mean inhibition (%)** | **SD (%)** |
| --- | --- | --- | --- |
| Bethlehem-fresh | 0.333 | 2.85 | 0.15 |
| Bethlehem-fresh | 3.333 | 4.11 | 1.14 |
| Bethlehem-fresh | 8.333 | 9.99 | 1.79 |
| Bethlehem-fresh | 16.667 | 17.38 | 2.16 |
| Bethlehem-fresh | 25.000 | 26.78 | 2.33 |
| Bethlehem-fresh | 33.333 | 31.15 | 0.58 |
| Bethlehem-dry | 0.333 | 1.85 | 0.29 |
| Bethlehem-dry | 3.333 | 3.11 | 0.73 |
| Bethlehem-dry | 8.333 | 9.32 | 0.44 |
| Bethlehem-dry | 16.667 | 16.88 | 0.26 |
| Bethlehem-dry | 25.000 | 23.09 | 0.39 |
| Bethlehem-dry | 33.333 | 28.21 | 0.76 |
| Hebron-fresh | 0.333 | 1.91 | 1.12 |
| Hebron-fresh | 3.333 | 6.09 | 0.33 |
| Hebron-fresh | 8.333 | 13.09 | 0.75 |
| Hebron-fresh | 16.667 | 20.95 | 1.21 |
| Hebron-fresh | 25.000 | 27.25 | 0.86 |
| Hebron-fresh | 33.333 | 34.40 | 0.85 |
| Hebron-dry | 0.333 | 3.05 | 1.03 |
| Hebron-dry | 3.333 | 5.80 | 0.80 |
| Hebron-dry | 8.333 | 11.25 | 0.00 |
| Hebron-dry | 16.667 | 18.12 | 0.44 |
| Hebron-dry | 25.000 | 24.42 | 0.22 |
| Hebron-dry | 33.333 | 29.58 | 1.00 |
| Trolox | 0.333 | 7.02 | 0.56 |
| Trolox | 1.000 | 23.17 | 0.52 |
| Trolox | 1.667 | 40.51 | 0.91 |
| Trolox | 2.333 | 55.98 | 1.35 |
| Trolox | 3.333 | 81.39 | 1.35 |
| Trolox | 5.000 | 97.08 | 0.98 |
| Trolox | 10.000 | 98.51 | 0.13 |

Values are calculated from three independent replicate absorbance measurements. SD represents the standard deviation among the three replicate measurements.

**Supplementary Table S4. DPPH control absorbance values**

| **Sample group** | **DPPH control absorbance at 517 nm** |
| --- | --- |
| Bethlehem-fresh | 0.397 |
| Bethlehem-dry | 0.397 |
| Hebron-fresh | 0.471 |
| Hebron-dry | 0.471 |
| Trolox | 0.446 |

**Calculation procedure**

For each replicate, percentage DPPH radical inhibition was calculated according to:

**% inhibition = [(A_control − A_sample)/A_control] × 100**

where *A_control* is the absorbance of the DPPH control at 517 nm and *A_sample* is the absorbance measured for the corresponding essential-oil or Trolox replicate.

For each concentration, the three replicate percentage-inhibition values were used to calculate the arithmetic mean and standard deviation (SD). No fixed coefficient of variation was imposed on the data.

Because 50% inhibition was not achieved by the essential-oil samples within the experimentally tested concentration range of 0.333–33.333 µg/mL, IC₅₀ values were not calculated or extrapolated from these data.

**Supplementary Table S5. One-way ANOVA of DPPH radical-scavenging activity at 33.333 µg/mL**

The DPPH inhibition values at the highest experimentally tested concentration (33.333 µg/mL) were compared among the four *C. capitatus* essential-oil samples using one-way analysis of variance (ANOVA), followed by Tukey's multiple-comparison test.

| **Source of variation** | **df** | **Sum of squares** | **Mean square** | **F** | **p-value** |
| --- | --- | --- | --- | --- | --- |
| Between groups | 3 | 68.62 | 22.87 | 35.49 | <0.001 |
| Within groups (Error) | 8 | 5.80 | 0.72 | — | — |
| Total | 11 | 74.42 | — | — | — |

**Post-hoc Tukey multiple-comparison test:**
Hebron fresh differed significantly from Bethlehem fresh, Hebron dry, and Bethlehem dry (*p* < 0.05). Bethlehem fresh differed significantly from Hebron dry and Bethlehem dry (*p* < 0.05). Hebron dry and Bethlehem dry did not differ significantly (*p* > 0.05).

Therefore, the statistical grouping was:

| **Sample** | **Mean ± SD (%)** | **Tukey group** |
| --- | --- | --- |
| Hebron fresh | 34.40 ± 0.85 | a |
| Bethlehem fresh | 31.15 ± 0.58 | b |
| Hebron dry | 29.58 ± 1.00 | c |
| Bethlehem dry | 28.21 ± 0.76 | c |
