# Supplementary material for: Essential oil composition and biological activities of Coridothymus capitatus collected from two locations in Palestine
Source: PLoS One. 2026 Sep 2;21(9):e0357443. doi: 10.1371/journal.pone.0357443 (PMC13537545; doi:10.1371/journal.pone.0357443)
Supplement: Supplementary file 2 [file pone.0357443.s004.docx]

PONE-D-26-11174

Essential Oil Composition and Biological Activities of Coridothymus capitatus Collected from Two Locations in Palestine

PLOS One

Dear Dr. Bsharat,

Thank you for submitting your manuscript to PLOS ONE. After careful consideration, we feel that it has merit but does not fully meet PLOS ONE’s publication criteria as it currently stands. Therefore, we invite you to submit a revised version of the manuscript that addresses the points raised during the review process.

Please consider the suggestions of both reviewers

Response: We sincerely thank the Editor and the reviewers for their valuable comments and constructive suggestions. We have carefully addressed all comments and revised the manuscript accordingly. A detailed, point-by-point response is provided below, and all changes have been incorporated into the revised manuscript.

We appreciate your time and consideration and hope that the revised manuscript is now suitable for publication in **PLOS ONE**.

Reviewer #1: General Evaluation
The manuscript investigates the chemical composition and biological activities of essential oils (EOs) extracted from Coridothymus capitatus collected from two Palestinian regions. The topic is of great interest due to the increasing interest in natural antimicrobial and antioxidant agents. The study combines GC-MS profiling with antioxidant, antibacterial, and antifungal assays, which provides useful preliminary data.
However, the manuscript currently has several methodological, analytical, and presentation weaknesses that should be addressed before publication. Major revisions are recommended.

Major Comments
**Introduction**
Novelty and Scientific Contribution Need Better Clarification
The manuscript largely confirms already established properties of C. capitatus EO (high thymol/carvacrol content and antimicrobial activity). The authors should better explain, what is genuinely novel in this study, why the Palestinian plant populations are scientifically important, and how this work advances beyond previous Mediterranean chemotype studies. The Introduction should explicitly define the research gap. Currently, the rationale remains descriptive rather than hypothesis-driven.

Response: We thank the reviewer for this insightful comment. We agree that the study's originality and scientific importance should be better communicated. The introduction has been considerably revised to clarify the research gap, study hypothesis, and scientific value of studying Palestinian Coridothymus capitatus communities.

We note that previous research has focused on C. capitatus populations from multiple Mediterranean states, using diverse sample sites, phenological phases, extraction procedures, and biological assays, making direct comparisons difficult. Geography has been shown to affect C. capitatus essential oils, but no study has examined the synergistic effects of geographical origin and post-harvest drying on the chemical composition and biological activities of essential oils from different Palestinian populations under uniform experimental conditions.

The Introduction now states that the Hebron and Bethlehem regions' different environmental conditions and post-harvest drying affect volatile secondary metabolite biosynthesis, resulting in significant differences in essential oil composition and biological activities. Instead of verifying C. capitatus essential oil's antioxidant and antibacterial characteristics, this study presents the first systematic comparative examination of fresh and dried leaves from two geographically distinct Palestinian groups. This study integrates chemical characterization with antioxidant, antibacterial, and antifungal property assessments in a single experimental framework to reduce methodological variability and directly evaluate the effects of geographical origin and drying. These findings help us understand Palestinian C. capitatus chemotype diversity and Mediterranean populations of this therapeutic species.

The introduction's final paragraph includes the changes.

**Materials & methods**
Lines 133-135 The description of the plant material is unclear. The authors refer here to “fresh” and “dried” leaves; however, the manuscript does not clearly explain earlier in the Methods section that two different leaf conditions/treatments were included in the experimental design. This should be explicitly clarified before presenting the extraction yields in order to avoid confusion for the reader.

**Response:** Thank you for this valuable comment. We have revised the Materials and Methods section to clarify the experimental design. Specifically, we now explicitly state in Section 2.1 that the collected leaves from each location were divided into two portions: one portion was used immediately for essential oil extraction as fresh material, while the other portion was shade-dried and subsequently used for extraction. This clarification has been incorporated before the extraction procedure to avoid confusion.

Line 182 The use of phenol and bleach as positive controls for antifungal activity is unusual and insufficiently justified. Standard antifungal agents (e.g., amphotericin B, nystatin, fluconazole, or commercial fungicides) would be more appropriate for comparison and would improve the scientific validity and comparability of the results. The authors should justify their choice of controls or reconsider the experimental design.

**Response:** We thank the reviewer for highlighting this important point. We agree that amphotericin B, nystatin, fluconazole or an established commercial fungicide would be more conventional reference chemicals and allow comparison with other antifungal investigations.

Phenol and sodium hypochlorite were selected as widely accessible disinfectants and are utilized in sanitation techniques for the disinfection of tools, equipment and contaminated materials to prevent microbial contamination. They were included to provide a locally relevant and practical benchmark for comparison with the tested plant extracts rather than to represent standard clinical antifungal agents.

The fungal isolates of this study are phytopathogenic fungi isolated and identified from diseased Palestinian plants. The clinical antifungal drugs (e.g., amphotericin B, nystatin and fluconazole) are mainly used for the medically important fungi, not to be used routinely as reference compounds for evaluation of plant pathogenic fungi. Furthermore, a lot of investigations on plant extracts have used phenol and/or sodium hypochlorite as broad-spectrum antibacterial reference disinfectants and not as typical antifungal medications. Therefore, we have edited the text to state that phenol and sodium hypochlorite were used as reference disinfectant controls rather than standard antifungal controls and have stated this as a limitation of the current work.

[Rogawansamy S, Gaskin S, Taylor M, Pisaniello D. An evaluation of antifungal agents for the treatment of fungal contamination in indoor air environments. Int J Environ Res Public Health. 2015 Jun 2;12(6):6319-32. doi: 10.3390/ijerph120606319. PMID: 26042369; PMCID: PMC4483703.]

**Copes WE, Ojiambo PS.** 2021. *Efficacy of Hypochlorite as a Disinfestant Against Fungal Pathogens in Agricultural and Horticultural Plant Production: A Systematic Review and Meta-Analysis.* **Phytopathology** 111(8):1369–1379. <https://doi.org/10.1094/PHYTO-05-20-0201-R>.

**Copes WE, Ojiambo PS.** 2021. *Efficacy of Hypochlorite in Disinfesting Nonfungal Plant Pathogens in Agricultural and Horticultural Plant Production: A Meta-Analysis.* **Plant Disease** 105:4084–4094. https://doi.org/10.1094/PDIS-09-20-2046-RE.

**Gupta AK, Ahmad I, Summerbell RC.** 2002. *Fungicidal activities of commonly used disinfectants and antifungal pharmaceutical spray preparations against clinical strains of Aspergillus and Candida species.* **Medical Mycology** 40:201–208.

Line 185-187 The determination of MIC is insufficiently described. The authors state that fungal growth inhibition was evaluated “visually or by quantifying turbidity/optical density,” but it is unclear which method was ultimately used for each experiment. The methodology should be standardized and described more precisely, including the wavelength used for OD measurements, cutoff criteria for inhibition, and whether measurements were performed spectrophotometrically or by visual inspection alone.

**Response:** The methodology was modified after preliminary observations showed that measuring optical density at 600 nm (OD600) was not a reliable indicator of microbial growth in the presence of the oil treatments. The oils caused significant optical interference, producing background artifacts that affected absorbance readings and prevented accurate differentiation between microbial growth and the intrinsic turbidity of the oil. Consequently, OD600 measurements could not be used as a dependable method for assessing growth inhibition. Instead, each well was examined individually using an ocular dissecting microscope to directly assess the presence or absence of visible fungal mycelial growth and bacterial turbidity. This direct visual evaluation provided a more accurate and reproducible assessment of microbial growth, eliminating the interference caused by the oils and allowing for reliable determination of antimicrobial activity.

Statistical analysis

The statistical section is insufficient. The authors don’t mention which type of ANOVA, no post hoc test is reported, no p-values are shown, how many were the replicates? The manuscript requires substantially improved statistical treatment.

**Response:** All experiments were performed in triplicate (n = 3), and the results are presented as the mean ± standard deviation (SD). IC₅₀ values were calculated by plotting the percentage of DPPH radical scavenging activity against sample concentration using Microsoft Excel. Statistical analyses were performed using one-way analysis of variance (one-way ANOVA), followed by Tukey's multiple comparison post hoc test to determine significant differences among the means. Differences were considered statistically significant at p < 0.05. This has been added and clarified in section 2.6. **For** Minimum inhibitory concentration (MIC) values were determined as discrete two-fold serial dilution endpoints (e.g., 25, 50, or 100 µg/mL) rather than continuous quantitative measurements. Consequently, conventional inferential statistical analyses are generally not appropriate for comparing MIC values among treatments. To ensure the reliability and reproducibility of the results, all experiments were performed independently in triplicate, and identical MIC values were obtained in all replicates. Therefore, the MIC values are reported directly without statistical comparison, which is consistent with standard practice in antimicrobial susceptibility testing and MIC determination studies

**Results & Discussion**
GC-MS Identification Requires More Rigor
Lines 196-207 The GC-MS section currently lacks critical analytical details required for reproducibility and validation. The authors must clarify whether authentic standards were used for compound verification. Additionally, please specify the exact version of the mass spectral library used and the minimum similarity threshold accepted for tentative identification. It is also unclear whether Kovats retention indices were experimentally validated; this must be explicitly stated.

**Response:** We thank the reviewer for this valuable comment. To improve the reproducibility and transparency of the GC–MS analysis, we have revised the Materials and Methods section (section 2.3) by providing additional details regarding compound identification. The EO constituents were identified using a combination of **authentic reference standards, mass spectral library matching, and retention index comparison. 15 authentic reference compounds,** including the major constituents of Coridothymus capitatus essential oil, were analyzed under identical chromatographic conditions to verify compound identities by comparing both their retention times and mass spectra with those of the sample constituents. The remaining compounds were tentatively identified by comparison of their mass spectra with those contained in the **NIST/EPA/NIH Mass Spectral Library (NIST 17)** and by comparison of their experimentally determined Kovats retention indices with published literature values obtained on the same stationary phase. A **minimum spectral similarity score of 90%** was used as the criterion for tentative identification, while compounds with lower similarity scores were further evaluated based on their fragmentation patterns and retention behavior before assignment. The Kovats retention indices were experimentally determined using a homologous series of **n-alkanes** analyzed under the same chromatographic conditions as the essential oil samples and were compared with published retention index data to further confirm compound identities.

Additionally, we use published retention index data reported by **Adams (2007)**; Adams RP. Identification of Essential Oil Components by Gas Chromatography/Mass Spectrometry. 4.1 ed. Carol Stream, IL, USA: Allured Publishing Corporation; 2007.

Regarding the data presentation, there are several major flaws in Table 1. Listing numerous compounds as “ND” without proper identification is insufficient; unknown peaks must either be identified, discussed if they are biologically relevant, or omitted entirely if they fall below the significance threshold. Finally, placeholder notation such as “??” is unacceptable in a finalized manuscript and must be corrected.

**Response:** We thank the reviewer for this helpful suggestion. To improve the clarity and readability of the manuscript, the original table has been replaced with a new table summarizing the identified compounds according to their chemical classes (e.g., monoterpene hydrocarbons, oxygenated monoterpenes, sesquiterpene hydrocarbons, oxygenated sesquiterpenes, and phenylpropanoids) In addition, compounds that were not identified or occurred only in trace amounts (<0.1%) have been omitted from the table, except for a few compounds present above this threshold that were retained for completeness as suggested. This presentation provides a clearer overview of the essential oil composition and facilitates comparison of the major chemical classes among the studied samples.

**Minor Comments**
Inconsistency in Geographic Locations
There is inconsistency regarding collection sites, most sections refer to Hebron and Bethlehem, but Tables 3 and 4 mention “Ramallah.” Please correct throughout the manuscript.

**Response:** Thank you for pointing out this inconsistency. We have carefully reviewed the manuscript and corrected the geographic locations throughout. The incorrect mention of "Ramallah" in Tables 3 and 4 has been replaced with the correct collection sites, "Hebron" and "Bethlehem," ensuring consistency across the entire manuscript.

Table Presentation
Table 1 is overcrowded and difficult to read. Consider, separating major/minor constituents, grouping by terpene class, or moving full chromatographic details to supplementary material.

**Response:** We thank the reviewer for this valuable suggestion. To improve the readability of Table 1, we have revised its presentation by grouping the identified constituents according to their terpene classes (monoterpene hydrocarbons, oxygenated monoterpenes, sesquiterpene hydrocarbons, oxygenated sesquiterpenes, and others). In addition, compounds that were not identified or occurred only in trace amounts (<0.1%) have been omitted from the table, except for a few compounds present above this threshold that were retained for completeness. As a result, the revised table is substantially less crowded while preserving all information necessary for interpretation of the chemical composition. We believe that, in its revised form, Table 1 is sufficiently concise and therefore have retained it in the main manuscript rather than moving it to the Supplementary Material.

**Question: Table 3 and table 4 lacks SD**

**Response: Concerning Table 3 and 4:**

Minimum inhibitory concentration (MIC) values were determined as discrete two-fold serial dilution endpoints (e.g., 25, 50, or 100 µg/mL) rather than continuous quantitative measurements. Consequently, conventional inferential statistical analyses are generally not appropriate for comparing MIC values among treatments. To ensure the reliability and reproducibility of the results, all experiments were performed independently in triplicate, and identical MIC values were obtained in all replicates. Therefore, the MIC values are reported directly without statistical comparison, which is consistent with standard practice in antimicrobial susceptibility testing and MIC determination studies

References
Finally, the reference list appears outdated. The authors should revise and update their citations to include recent relevant literature, ensuring the discussion aligns with current developments.

**Response:** We appreciate the reviewer's valuable suggestion. In response, we thoroughly revised the reference list and updated it with recent and relevant publications, particularly those published between 2022 and 2024. Several older or less relevant references were replaced with recent reviews and original research articles that better reflect current knowledge on *Coridothymus capitatus* (*Thymbra capitata*), essential oil chemistry, biological activities, antimicrobial properties, and bioherbicidal applications. The Discussion section was also revised where appropriate to incorporate these updated references and ensure that the manuscript is aligned with the latest developments in the field.

Reviewer #2: Methodological concern — statistical analysis and replication
The Methods section (2.6) states that data are expressed as mean ± SEM or mean ± SD, and that ANOVA was used to assess differences. However, the number of biological and technical replicates is never specified for any assay. It is impossible to evaluate the validity of the reported means or the reliability of statistical comparisons without this information. The authors must clarify: (a) how many independent extractions were performed per sample type; (b) how many replicate wells/measurements were used per assay; and (c) whether the ANOVA results (including post-hoc tests and p-values) can be provided in tables or supplementary material. As currently written, Table 2 presents IC₅₀ ± values that appear to be SEM but lack statistical group assignments, and Tables 3–4 report single MIC values with no measure of variability or indication that assays were repeated.

**Response:**

a. **Response:**
   We thank the reviewer for this important comment. **Three independent hydrodistillation extractions were performed for each sample type (fresh and dried leaves collected from Hebron and Bethlehem).** This information has now been added to the Materials and Methods section of the revised manuscript to improve clarity in section 2.2.
b. **Response:** We thank the reviewer for this important comment. **All antioxidant, antibacterial, and antifungal assays were performed in triplicate. For the broth microdilution assays, each concentration was tested in three replicate wells, and the entire experiment was independently repeated three times. The reported results represent the consistent outcomes obtained across these independent experiments.** This information has now been added to the Materials and Methods section (section 2.5).
c. **Response:** All experiments were performed in triplicate (n = 3), and the results are presented as the mean ± standard deviation (SD). IC₅₀ values were calculated by plotting the percentage of DPPH radical scavenging activity against sample concentration using Microsoft Excel. Statistical analyses were performed using one-way analysis of variance (one-way ANOVA), followed by Tukey's multiple comparison post hoc test to determine significant differences among the means. Differences were considered statistically significant at p < 0.05. This has been added and clarified in section 2.6 **For** Minimum inhibitory concentration (MIC) values were determined as discrete two-fold serial dilution endpoints (e.g., 25, 50, or 100 µg/mL) rather than continuous quantitative measurements. Consequently, conventional inferential statistical analyses are generally not appropriate for comparing MIC values among treatments. To ensure the reliability and reproducibility of the results, all experiments were performed independently in triplicate, and identical MIC values were obtained in all replicates. Therefore, the MIC values are reported directly without statistical comparison, which is consistent with standard practice in antimicrobial susceptibility testing and MIC determination studies

Figure 1 — critical deficiencies
The dose–response figure for the DPPH assay has several serious problems. First, the x-axis is labeled "Conc" without units, which is unacceptable. Second, the concentration range shown (up to ~33 µg/mL) does not reach the reported IC₅₀ values for the EOs (61–70 µg/mL), meaning the curves for the four EO samples never approach 50% inhibition within the plotted range. This makes IC₅₀ determination from the figure impossible to verify and raises doubts about whether the values in Table 2 were obtained by interpolation, extrapolation, or curve fitting. Third, the figure title refers to "Zohif," a term that does not appear anywhere else in the manuscript and appears to be an artifact of a different experiment or a translation error. Fourth, axis labels and legend font sizes are too small. The figure must be entirely rebuilt with appropriate axes, units, full concentration range (at least up to 100 µg/mL as stated in the methods), and a corrected title.

**Response:**

First, the x-axis is labeled "Conc" without units, which is unacceptable.

We thank the reviewer for this valuable comment. The x-axis label has been corrected by replacing "Conc" with **"Conc (μg/mL)"**, thereby including the appropriate unit of concentration. The revised figure now clearly presents the x-axis as **Conc (μg/mL)**.

Second, the concentration range shown (up to ~33 µg/mL) does not reach the reported IC₅₀ values for the EOs (61–70 µg/mL), meaning the curves for the four EO samples never approach 50% inhibition within the plotted range. This makes IC₅₀ determination from the figure impossible to verify and raises doubts about whether the values in Table 2 were obtained by interpolation, extrapolation, or curve fitting.

**Response:**

Base on the figure 1 that we calculated based on experimental test we concluded as the Trolox had (IC₅₀ = 4.73±0.23 μg/ml) exhibited the strongest antioxidant activity, as expected for the standard reference antioxidant. Its low IC₅₀ value indicates high free radical scavenging efficiency. Meanwhile the curves for the four EO samples never approach 50% inhibition within the plotted range. Yes; this makes IC₅₀ determination from the figure impossible to verify. Therefore, the values in Table 2 were estimated by extrapolation.

Third, the figure title refers to "Zohif," a term that does not appear anywhere else in the manuscript and appears to be an artifact of a different experiment or a translation error.

**Response:** We thank the reviewer for identifying this error. The term "Zohif" was an inadvertent labeling mistake. It has been corrected and replaced with "*Coridothymus capitatus*" throughout the figure to ensure consistency with the manuscript.

Fourth, axis labels and legend font sizes are too small. The figure must be entirely rebuilt with appropriate axes, units, full concentration range (at least up to 100 µg/mL as stated in the methods), and a corrected title.

**Response:** We thank the reviewer for this helpful suggestion. The figure has been completely revised. Axis labels and legend font sizes have been increased to improve readability, and the figure has been rebuilt with appropriate axes, units, and a corrected title. In addition, the concentration range has been updated to fully reflect the experimental design (up to 33 µg/mL). The discussion section has also been revised to include the correct effective concentration value (33 µg/mL) for consistency with the results.

Inconsistency in Table 3 and 4 captions vs. collection sites
Both Table 3 and Table 4 captions state that the EOs were "collected from Ramallah in Palestine." This is incorrect — the study explicitly states material was collected from Hebron and Bethlehem. This is not a minor typo; it suggests that tables may have been recycled or copied from a different dataset, which undermines confidence in the data integrity. The authors must correct this and confirm that the data in both tables correspond to the Hebron and Bethlehem samples described throughout the manuscript.

**Response:** Thank you for pointing out this inconsistency. We have carefully reviewed the manuscript and corrected the geographic locations throughout. The incorrect mention of "Ramallah" in Tables 3 and 4 has been replaced with the correct collection sites, "Hebron" and "Bethlehem," ensuring consistency across the entire manuscript.

Comparison with positive controls in antifungal assays (Table 4)
The authors state that the EOs showed "much superior antifungal efficacy" compared to phenol and 3% bleach. However, phenol and bleach are biocidal disinfectants, not standard clinical or agricultural antifungal agents. Their use as positive controls is scientifically inappropriate for an antifungal MIC assay; recognized positive controls such as fluconazole, amphotericin B, or imazalil are expected. Furthermore, the MIC values reported for phenol (3,516 µg/mL) and bleach (1,875 µg/mL) are dramatically higher than those for the EOs, which is not a meaningful comparison and could mislead readers. The authors should either replace these controls with appropriate antifungal agents or provide a clear pharmacological justification for the choice and refrain from drawing superiority conclusions from this comparison.

**Response:** We thank the reviewer for highlighting this important point. We agree that amphotericin B, nystatin, fluconazole or an established commercial fungicide would be more conventional reference chemicals and allow comparison with other antifungal investigations.

Phenol and sodium hypochlorite were selected as widely accessible disinfectants. They were included to provide a locally relevant and practical benchmark for comparison with the tested plant extracts rather than to represent standard clinical antifungal agents.

The fungal isolates of this study are phytopathogenic fungi isolated and identified from diseased Palestinian plants. The clinical antifungal drugs (e.g., amphotericin B, nystatin and fluconazole) are mainly used for the medically important fungi, not to be used routinely as reference compounds for evaluation of plant pathogenic fungi. Furthermore, a lot of investigations on plant extracts have used phenol and/or sodium hypochlorite as broad-spectrum antibacterial reference disinfectants and not as typical antifungal medications. Therefore, we have edited the text to state that phenol and sodium hypochlorite were used as reference disinfectant controls rather than standard antifungal controls and have stated this as a limitation of the current work.

[Rogawansamy S, Gaskin S, Taylor M, Pisaniello D. An evaluation of antifungal agents for the treatment of fungal contamination in indoor air environments. Int J Environ Res Public Health. 2015 Jun 2;12(6):6319-32. doi: 10.3390/ijerph120606319. PMID: 26042369; PMCID: PMC4483703.]

**Copes WE, Ojiambo PS.** 2021. *Efficacy of Hypochlorite as a Disinfestant Against Fungal Pathogens in Agricultural and Horticultural Plant Production: A Systematic Review and Meta-Analysis.* **Phytopathology** 111(8):1369–1379. <https://doi.org/10.1094/PHYTO-05-20-0201-R>.

**Copes WE, Ojiambo PS.** 2021. *Efficacy of Hypochlorite in Disinfesting Nonfungal Plant Pathogens in Agricultural and Horticultural Plant Production: A Meta-Analysis.* **Plant Disease** 105:4084–4094. https://doi.org/10.1094/PDIS-09-20-2046-RE.

**Gupta AK, Ahmad I, Summerbell RC.** 2002. *Fungicidal activities of commonly used disinfectants and antifungal pharmaceutical spray preparations against clinical strains of Aspergillus and Candida species.* **Medical Mycology** 40:201–208.

For Paecilomyces niveus, two EO samples (Hebron fresh and Hebron dry) showed "no effect" at the tested concentrations, yet no upper limit or ">100 µg/mL" designation is provided. The authors should report this as a defined MIC (e.g., >100 µg/mL) and discuss the biological significance of this resistance pattern, given that P. niveus is a known mycotoxin producer. The incomplete inhibition for specific samples also warrants further discussion beyond the single sentence currently offered.

**Response:**

For *Paecilomyces niveus,* the essential oils from Hebron fresh and Hebron dry exhibited no complete inhibition at the highest concentration tested (100 µg/mL), may reflect strain-specific physiological characteristics and/or differences in the chemical composition of the oils, particularly the relative abundance of bioactive constituents. These points have now been incorporated into the revised manuscript.

References [5] and [6] in the Introduction cite studies on Juniperus essential oils (Baser et al., 1994; Kokkini et al., 2004), yet they are cited in the context of C. capitatus biology and traditional medicine uses. This is an apparent error — either the wrong references were cited or the citation numbering was shifted during manuscript preparation. All references must be verified for accuracy and relevance.

**Response:** We thank the reviewer for this important observation. The issue was identified as a citation mismatch during manuscript revision. Accordingly, the incorrect references to *Juniperus* species have been removed and replaced with appropriate and relevant studies specifically related to *Coridothymus capitatus*. All citations have been carefully verified and renumbered to ensure accuracy, consistency, and relevance throughout the manuscript.

EO yields are reported (1.1–2.1%) but moisture content of the plant material was not assessed, as acknowledged by the authors. This omission weakens the interpretation of yield differences between fresh and dried samples, since yield on a fresh-weight basis is inherently confounded by water content. At minimum, the authors should provide the approximate moisture content estimated from the weight difference before and after drying, or acknowledge this as a quantitative limitation in both the Methods and Discussion.

Response: We thank the reviewer for this important comment. We agree that the absence of direct moisture content measurement limits strict quantitative comparison between fresh and dried plant material. We have now clarified this limitation in both the Methods and Discussion sections. Since fresh weight yield comparisons are inherently influenced by water content, we have revised the text to avoid over-interpretation of yield differences and explicitly acknowledge this as a methodological limitation. We couldn’t measure the moisture content of the sample because we haven’t the suitable tool for that at our university.

Minor Comments

The running title on the manuscript cover page reads "ssential Oil Composition and Biological Activities of Coridothymus capitatus" — the leading "E" is missing. This must be corrected.

**Response:** We thank the reviewer for pointing out this typographical error. The running title has been corrected to “Essential Oil Composition and Biological Activities of Coridothymus capitatus.”

Throughout the manuscript, "Bethlehem" is occasionally misspelled as "Beitlehem" (e.g., Table 1 footnote). All place names should be checked and standardized.

**Response:** Thank you for pointing this out. The spelling of “Bethlehem” has been checked throughout the manuscript and all occurrences, including Table 1 footnote, have been corrected and standardized.

Section 3.3 (Antibacterial Activity) contains a near-verbatim repetition of two consecutive paragraphs (lines 313–320 and 321–329), describing essentially the same results for B. cereus and S. aureus twice. One of these paragraphs should be removed.

Response: We thank the reviewer for this careful observation. We agree that Section 3.3 contained redundant repetition of the antibacterial results for *Bacillus cereus* and *Staphylococcus aureus*. The duplicated paragraph has been removed, and the section has been revised for clarity and conciseness.

The term "subjectively" in 2.3 ("identified both subjectively and quantitatively") is likely intended to mean "qualitatively." Please correct.

Response: We thank the reviewer for this observation. The term “subjectively” in Section 2.3 has been corrected to “qualitatively” to improve clarity and accuracy throughout the manuscript.

The DMSO solvent control is mentioned in 2.5 (final concentration 5%) but no results for this control appear in the results section. A statement confirming the absence of inhibitory effects at this DMSO concentration should be included.

**Response:**

The statement was added to the section 2.5

The DMSO as a solvent showed no inhibitory effect on the growth of the tested microorganisms at concentrations of 20% or lower, according to our previous disc diffusion experiments.

For the antibacterial MIC assay, only broth microdilution method reference [26] is cited; however, reference [26] (Hammer et al., 2003) describes antifungal methods for tea tree oil components, not a general antibacterial broth microdilution standard. The appropriate CLSI or EUCAST guideline should be cited instead.

**Response:**

We thank the reviewer for this important correction. We agree that the previously cited reference (Hammer et al., 2003) is not a standard guideline for antibacterial MIC determination. The manuscript has been corrected by replacing it with the appropriate CLSI (Clinical and Laboratory Standards Institute) broth microdilution guideline reference, and the Methods section has been updated accordingly.

The Conclusion mentions that "drying affected both chemical composition and bioactivity, typically augmenting antibacterial potency." This is an overgeneralization — the antifungal data show a mixed picture (e.g., Hebron fresh outperforms Hebron dry against Botrytis californica). The conclusion should more accurately reflect the nuanced results.

We appreciate the reviewer's insightful perspective. We agree that the impact of post-harvest drying on the biological activities of the EOs was overvalued in the original conclusion. While drying typically improved the antibacterial activity, especially against Gram-positive bacteria, its effect on antifungal activity diverse depending on the species and the EOs geographical origin. For instance, fresh oil from the Hebron population was more active against *Botrytis californica* than the corresponding dried oil, but drying had no effect or resulted in inconsistent reactions for other fungal species. The Conclusion has been updated to properly signify these findings by stressing that, rather than continuously increasing bioactivity, post-harvest drying altered the chemical composition and affected biological activities in a compound- and microorganism-dependent manner.

The Declarations section lists "Finding" instead of "Funding." Please correct.

**Response:** Thank you for pointing this out. The “Declarations” section has been corrected, and “Finding” has been replaced with the correct term “Funding.”
