# Supplementary material for: Essential oil composition and biological activities of Coridothymus capitatus collected from two locations in Palestine
Source: PLoS One. 2026 Sep 2;21(9):e0357443. doi: 10.1371/journal.pone.0357443 (PMC13537545; doi:10.1371/journal.pone.0357443)
Supplement: Supplementary file 3 [file pone.0357443.s005.docx]

PONE-D-26-11174R1

Essential Oil Composition and Biological Activities of Coridothymus capitatus Collected from Two Locations in Palestine

PLOS One

Dear Dr. Bsharat,

Thank you for submitting your manuscript to PLOS ONE. After careful consideration, we feel that it has merit but does not fully meet PLOS ONE’s publication criteria as it currently stands. Therefore, we invite you to submit a revised version of the manuscript that addresses the points raised during the review process.

**Please consider the suggestions done by the reviewer.**

**Response:** Thank you for the reviewer’s suggestions. We have carefully considered and addressed all of the comments raised by the reviewer and revised the manuscript accordingly. All relevant changes have been incorporated into the revised manuscript.

**Reviewer #1:** The authors have addressed all comments and the manuscript has been significantly improved. Thus, I think it is suitable for publication.

Response: We sincerely thank the reviewer for carefully evaluating our revised manuscript and for the positive assessment. We greatly appreciate the reviewer’s constructive comments and suggestions throughout the review process, which helped us improve the clarity, accuracy, and overall quality of the manuscript. We are grateful for the reviewer’s recommendation that the manuscript is suitable for publication.

**Reviewer #2:** Thank you for a detailed and courteous response. Several points have been resolved well — the restructuring of Table 1, the correction of the collection sites, the removal of the duplicated antibacterial paragraph, the replacement of the MIC methodology reference, and the more measured Conclusion regarding post-harvest drying. The comments below concern issues that remain open, together with some that emerged from the revision itself.

Major comments

IC₅₀ determination (Table 2, Fig. 1, Abstract, §3.2).
In your response you state that the Table 2 values were estimated by extrapolation. The assay spans 0.25–33 µg/mL, where the oils reach approximately 28–34% inhibition, while the reported IC₅₀ values are 61.16–70.55 µg/mL. Values obtained by projecting roughly twofold beyond the highest measured concentration cannot be reported to two decimal places with error terms, nor carried into the Abstract as findings, because the shape of the curve in the region of 50% inhibition has not been observed. Two routes are available:

(a) Preferred: repeat the DPPH assay over a range that reaches at least 100 µg/mL, so that 50% inhibition falls within the measured data, and report interpolated IC₅₀ values with the fitted model and R² stated.
(b) Acceptable alternative: withdraw the IC₅₀ values entirely and report percentage inhibition at the highest concentration tested (33 µg/mL) for each sample, with SD. This is a legitimate way to present the data you have, and the compositional comparison between chemotypes can still be made on that basis. The Abstract, §3.2, and the Conclusion would need to be revised accordingly, including the comparisons with the Moroccan, Libyan, and Tunisian studies, which are IC₅₀-based and would no longer be commensurable.

Please also state explicitly in §2.4 the concentration range actually assayed and the method used to derive IC₅₀ (linear regression, four-parameter fit, or other).

Response: Thank you for this helpful suggestion. We have revised the manuscript accordingly and have withdrawn the IC₅₀ values from the Abstract, Section 3.2, Table 2, and Conclusion. Instead, DPPH radical-scavenging activity is now reported as the percentage inhibition at the highest experimentally tested concentration (33.33 µg/mL), expressed as mean ± SD (n = 3), for each essential-oil sample.

We have also revised Section 2.4 to explicitly state that the DPPH assay was performed over the concentration range of 0.333–33.33 µg/mL. Because 50% inhibition was not consistently achieved across all essential-oil samples within this experimentally tested range, IC₅₀ values were not calculated or extrapolated. Consequently, no linear regression, four-parameter logistic model, or other IC₅₀-fitting method was used.

In addition, the IC₅₀-based comparisons with previously published Moroccan, Libyan, and Tunisian *C. capitatus*/*T. capitatus* essential oils have been removed from Section 3.2, since direct comparison between IC₅₀ values reported in those studies and percentage inhibition measured at 33.33 µg/mL in the present study would not be methodologically commensurable. The Abstract and Conclusion have likewise been revised to reflect this change.

Reported standard deviations (Table 2).
Each of the five SDs is exactly 5.00% of its mean (61.16 ± 3.1; 65.02 ± 3.25; 69.26 ± 3.46; 70.55 ± 3.52; 4.73 ± 0.23). Independent triplicate measurements do not yield a constant coefficient of variation across samples. Please recalculate the dispersion from the raw absorbance data and provide those data — all replicates, all concentrations, all samples — as supplementary material, consistent with your Data Availability statement.

Response: Thank you for identifying this important issue. We agree that the previously reported standard deviations were not appropriately derived from the independent replicate measurements. We have therefore completely recalculated the DPPH radical-scavenging results from the original raw absorbance measurements.

The revised analysis was performed using the individual absorbance values recorded at 517 nm for each replicate at each tested concentration. For each sample and concentration, the mean absorbance and standard deviation were calculated from the three independent replicate measurements. Percentage DPPH inhibition was then calculated for each replicate using the DPPH control absorbance according to the equation:

% inhibition = [(A_control − A_sample)/A_control] × 100.

The mean ± SD values were subsequently calculated from the three replicate inhibition values. Thus, the revised SDs are derived from the actual experimental replicate measurements and are no longer based on a fixed coefficient of variation.

We have also added the complete raw DPPH dataset as Supplementary Material. The supplementary file contains the individual absorbance measurements for all three replicates, all tested concentrations, all four *Coridothymus capitatus* essential-oil samples (Hebron-fresh, Hebron-dry, Bethlehem-fresh, and Bethlehem-dry), and the Trolox control. The corresponding calculated percentage-inhibition values are also provided to ensure complete transparency and reproducibility of the analysis.

In accordance with our revised analysis, the previously reported IC₅₀ values have been removed from the manuscript. Because 50% inhibition was not consistently achieved by all essential-oil samples within the experimentally tested concentration range, IC₅₀ values were not calculated or extrapolated. No linear regression, four-parameter logistic model, or other curve-fitting procedure was therefore used to derive IC₅₀ values.

Table 2 has been revised accordingly and now reports DPPH inhibition at the highest experimentally tested concentration (33.33 µg/mL) as mean ± SD based on the independent replicate measurements. The Abstract, Section 3.2, and Conclusion have also been revised to remove the previous IC₅₀-based statements.

The complete raw dataset and the corresponding calculations are provided in Supplementary Table S1.

Discrepancy between Figure 1 and Table 2.
The Bethlehem-fresh curve lies above the Hebron-fresh curve at every plotted concentration (approximately 34.5% versus 31% at 33 µg/mL), yet Table 2 reports Hebron fresh as the most active sample. Since §3.2 attributes the higher activity of Hebron fresh to its elevated thymol content, the interpretation depends on which source is correct. Please confirm the ranking and revise whichever presentation is in error. The Trolox curve is a separate concern: reaching approximately 81% inhibition at 3.3 µg/mL, it implies an IC₅₀ near 2 µg/mL rather than the tabulated 4.73 µg/mL.

**Response:** Thank you for identifying this discrepancy. We carefully rechecked the raw absorbance data and confirmed that the labels for the Bethlehem-fresh and Hebron-fresh samples had inadvertently been interchanged in the previous presentation. This labeling error has now been corrected. Based on the raw absorbance data, Hebron-fresh EO showed the highest DPPH radical-scavenging activity at the highest tested concentration (33.33 µg/mL), followed by Bethlehem-fresh, Hebron-dry, and Bethlehem-dry EOs, with values of 34.40 ± 0.85%, 31.15 ± 0.58%, 29.58 ± 1.00%, and 28.21 ± 0.76%, respectively. Figure 1, Table 2, and the corresponding discussion have been revised to reflect this corrected ranking. The statement linking the higher activity of Hebron-fresh EO to its chemical composition has also been retained only in the context of the corrected data and appropriately qualified.

We also rechecked the Trolox data against the original replicate absorbance measurements. The previously reported Trolox IC₅₀ value of 4.73 µg/mL was not consistent with the raw data and has therefore been removed. The raw data show 40.51 ± 0.91% inhibition at 1.667 µg/mL and 55.98 ± 1.35% inhibition at 2.333 µg/mL, indicating that 50% inhibition was reached between these concentrations. In accordance with the revised approach of avoiding IC₅₀ calculations when they are not necessary for the comparison, we have removed the previously reported Trolox IC₅₀ value rather than introducing a new fitted value. Trolox is now presented as a positive control based on its concentration-dependent DPPH radical-scavenging activity.

The revised manuscript and Supplementary Material now report the raw replicate absorbance measurements and the recalculated percentage inhibition values, allowing the reported results to be independently verified.

Magnitude of the MIC values.
Antibacterial MICs of 25–100 µg/mL and antifungal MICs as low as 6.25 µg/mL are one to three orders of magnitude below the range generally reported for thymol- and carvacrol-rich Lamiaceae essential oils, which typically falls between 0.25 and 5 mg/mL. Your DPPH results are displaced in the same direction relative to the comparator studies you cite. Please verify the calculation of final in-well concentrations and confirm that the 100 µL of inoculum added to each 100 µL of diluted oil has been accounted for in the reported values — this alone represents a twofold factor. Please also confirm the units (µg/mL versus µL/mL) and state how the oil mass was determined for the stock solution.

**Response:** Thank you for raising this important point. We re-examined the original experimental records and identified an error in the description of the EO stock-solution preparation in the revised manuscript. For each assay, 20 mg of EO was accurately weighed and diluted to a final volume of 100 mL to obtain a 200 µg/mL stock solution. The antibacterial stock solutions were prepared in Mueller–Hinton broth containing 20% (v/v) DMSO, whereas the antifungal stock solutions were prepared in RPMI 1640 medium containing 20% (v/v) DMSO.

Two-fold serial dilutions were prepared in the corresponding assay medium, producing EO concentrations of 100, 50, 25, 12.5, 6.25, 3.125, 1.563, 0.781, 0.391, and 0.195 µg/mL before inoculation. All dilutions were prepared aseptically in sterile 96-well microtiter plates. The maximum DMSO concentration was 10% (v/v) in the first well before inoculation and decreased proportionally with each subsequent two-fold dilution. Corresponding solvent controls containing equivalent DMSO concentrations without EO were included to determine whether DMSO affected microbial growth.

The description and concentration calculations have been corrected in the revised manuscript. We thank the reviewer for identifying this issue, which allowed us to improve the clarity and reproducibility of the antimicrobial assay.

Question: Undertakings not carried into the manuscript.
Four items promised in the response letter are absent from the revised text:
The superiority claim persists at lines 443–444, where the EOs are said to show "much greater antifungal efficacy at slightly reduced dosages." A difference of 6.25 versus 3516 µg/mL is roughly 560-fold, not slight, and the comparison is against controls you have now agreed are not antifungal agents. Please remove the comparative claim and simply report both sets of values.

Response: We acknowledge that it was misleading to call the EO concentrations “slightly reduced” and that direct comparison of efficacy with phenol and bleach was inappropriate since these were included only as practical disinfection reference controls, and not as conventional antifungal agents or agricultural fungicides. We have therefore deleted the statement that the EOs showed ‘much greater antifungal efficacy at slightly reduced dosages’ and all claims of comparative superiority.

The stated limitation regarding phenol and bleach as reference disinfectants does not appear in the text. Please add it to §2.5 and to the Discussion.

**Response:** Thank you for pointing out this omission. We have now explicitly stated the limitation associated with using phenol and commercial bleach as reference disinfectants in both Section 2.5 and the Discussion. We clarified that these substances are neither conventional antifungal agents nor agricultural fungicides and that their MIC values were included only as descriptive reference values under the conditions of the present assay. Consequently, they were not used to establish comparative superiority or equivalent efficacy of the EOs.

Table 4 still reports "no effect" for P. niveus. Please replace with ">100 µg/mL" in the table itself.

Response: Thank you for pointing this out. We have revised **Table 4** accordingly. The entries previously reported as “no effect” for *Paecilomyces niveus* have been replaced with **“>100 µg/mL”**, indicating that no inhibition was observed at the highest EO concentration tested (100 µg/mL).

No statistical group assignments, p-values, or post-hoc results appear. Having described one-way ANOVA with Tukey's test in §2.6, please add superscript letters to Table 2 denoting significantly different groups, report the relevant p-values, and provide the full ANOVA output as supplementary material.

**Response:** Thank you for this helpful comment. We have added statistical group assignments to Table 2 using superscript letters to indicate significant differences among the four essential-oil samples at 33.33 µg/mL. We also report the one-way ANOVA result (*F*(3,8) = 35.49, *p* < 0.001) and the corresponding Tukey multiple-comparison results. The complete ANOVA summary and Tukey grouping have been added to the Supplementary Material as Supplementary Table S5. The revised Table 2 and corresponding statistical description in the Results section have been updated accordingly.

Reference accuracy.
The response states that all citations were verified. The following remain incorrect:

[5] and [16] are the same publication (Saoulajan et al., Trends Food Sci Technol 2022;129:463–491), cited twice under different numbers.

Response: Thank you for identifying this error. We confirmed that References [5] and [16] were inadvertently duplicated. We have retained Reference [5] for the general background information on *C. capitatus* and replaced the duplicated Reference [16] with a more appropriate regional reference addressing the chemotypic variation of *C. capitatus* in Israel and the West Bank. The corresponding citation in the manuscript and the reference list have been corrected accordingly.

[14] (Roberts et al., bioherbicides for weed control) supports a sentence about Holy Land chemotype profiles.

**Response:** We thank the reviewer for identifying this citation issue. We agree that the original reference did not directly support the statement concerning the geographical variation in the chemical composition of *C. capitatus* essential oils. We have therefore replaced the inappropriate reference with El Ouariachi et al. (2011), which specifically investigated the chemical composition of *Thymus capitatus* essential oil and its biological properties. The corresponding sentence has also been revised to more accurately state that the chemical composition of *C. capitatus* essential oil can vary geographically, including differences in the relative abundance of major constituents such as thymol and carvacrol. The reference list and in-text citation have been corrected accordingly.

[37] (Tepe et al., Clinopodium extracts) and [38] (Lagouri & Boskou, spice screening) are cited for enzymatic conversion of γ-terpinene and p-cymene into thymol and carvacrol; neither addresses that pathway.

**Response:** Thank you for pointing this out. We agree that the previous references [37] and [38] did not directly support the specific statement concerning the biosynthetic conversion of γ-terpinene and p-cymene to thymol and carvacrol. We have therefore replaced these references with appropriate primary literature addressing the biosynthesis of phenolic monoterpenes. The text has also been revised to describe the pathway more accurately, emphasizing the experimentally supported role of γ-terpinene and the involvement of cytochrome P450 and short-chain dehydrogenase/reductase enzymes. The discussion of p-cymene has been qualified to avoid implying a definitively established enzymatic conversion pathway

[40] (Bhatt et al., doxycycline pharmacokinetics in Mycoplasma) is cited at line 340 for the antioxidant mechanism of thymol and carvacrol.

**Response:** Thank you for identifying this citation mismatch. We agree that the previously cited reference [40] (Bhatt et al., 2019) primarily addresses the pharmacokinetic/pharmacodynamic properties of doxycycline against *Mycoplasma hyopneumoniae* and does not directly support the general statement regarding its mechanism of antibacterial action. We have therefore replaced reference [40] with an appropriate reference addressing the molecular mechanism of tetracycline antibiotics, including inhibition of bacterial protein synthesis through interaction with the 30S ribosomal subunit. The corresponding sentence in the Discussion has also been revised for greater precision.
[35] — the author list does not correspond to Arch Biochem Biophys 187(2):307–314; please verify against the original.

**Response:** Thank you for identifying this bibliographic error. We rechecked reference [35] against the original publication and confirmed that the previously listed author names were incorrect. We have corrected reference [35] to the original authors, A. J. Poulose and R. Croteau, while retaining the correct journal, year, volume, issue, and page numbers. The corrected reference is now: Poulose AJ, Croteau R. *Biosynthesis of aromatic monoterpenes: conversion of γ-terpinene to p-cymene and thymol in Thymus vulgaris L.* Archives of Biochemistry and Biophysics. 1978;187(2):307–314. doi:10.1016/0003-9861(78)90039-5.

[27] is undated and cites M07-A10, a superseded edition (see m5 below).

**Response:** Thank you for pointing this out. We agree that the previously cited CLSI document M07-A10 was an outdated edition and that the reference was incomplete. We have replaced it with the current **12th edition of CLSI M07, published in 2024**, which supersedes the previous editions. The corresponding statement in §2.5 has also been revised to specify that the bacterial MIC determination was based on the broth microdilution procedures described in CLSI M07.

Please carry out a full citation-by-citation check rather than a spot correction.

**Response:** Thank you for the comment. I have carried out a full citation-by-citation check of the manuscript, rather than a spot correction. Each in-text citation was checked against the corresponding reference, and the references were revised where necessary to ensure consistency, relevance, and completeness.

Minor comments
Table 1 does not balance. The five class subtotals sum to exactly 100.00 in every column, while "Total identified" is given as 99.32–99.68. The difference equals the "Others" row in each case, indicating that "Others" represents the unidentified remainder — yet two identified compounds (nepetalactone, cis-carvyl propanoate) are listed under a heading "Other compounds." Please separate identified minor constituents from the unidentified fraction. Independently, the Hebron-fresh monoterpene hydrocarbons sum to 25.56 rather than the stated 25.68, and the oxygenated monoterpenes to 68.88 rather than 69.03. Please recheck all subtotals.

**Response:** We thank the reviewer for carefully checking the numerical consistency of Table 1. We have rechecked all compound-level percentages and recalculated the chemical-class subtotals. The previous “Others” category combined identified minor constituents (nepetalactone and cis-carvyl propanoate) with the unidentified fraction, which could incorrectly imply that these identified compounds were part of the unidentified remainder. We have therefore separated these components in the revised Table 1 by placing nepetalactone and cis-carvyl propanoate under **“Identified minor compounds”** and reporting the remaining unassigned percentage separately as **“Unidentified fraction.”**

We also corrected the class subtotals throughout the table. In particular, the monoterpene hydrocarbon and oxygenated monoterpene subtotals for the Hebron-fresh EO were corrected from 25.68% and 69.03% to **25.56% and 68.88%**, respectively. Additional rounding/subtotal discrepancies identified during the complete recheck were also corrected for the Hebron-dry EO. The revised table now distinguishes clearly between total identified constituents and the unidentified fraction, with the identified and unidentified fractions summing to **100.00%** for each EO sample.

We appreciate the reviewer’s observation, which has improved the numerical consistency and clarity of Table 1.

Nepetalactone. Its presence in a Thymbra/Coridothymus oil is unusual. Please confirm the identification against an authentic standard or a reported RI on the same stationary phase, or reclassify it as tentative.

**Response:** We thank the reviewer for highlighting the unusual occurrence of nepetalactone in *Thymbra/Coridothymus* essential oil. We agree that its identification should not be presented as definitive without confirmation using an authentic reference standard or a retention index determined on the same stationary phase. We have therefore revised Table 1 and now classify nepetalactone as a **tentatively identified compound**. The corresponding table footnote has been added to clarify that its identification was based on mass-spectral/library comparison and was not confirmed with an authentic reference standard. We have also revised the text describing the GC–MS identification procedure accordingly. This change avoids overstating the certainty of the compound identification.

Semi-quantification. The percentages appear to derive from TIC area normalisation without FID data or response factors. Please state this explicitly and describe the composition as semi-quantitative.

**Response:** We thank the reviewer for this important clarification. We confirm that the percentages reported in Table 1 were obtained by **TIC peak-area normalization of the GC–MS chromatograms**. No FID measurements, external calibration standards, or compound-specific response factors were used. Therefore, these values represent **semi-quantitative relative abundances** and should not be interpreted as absolute concentrations.

We have revised the manuscript accordingly. The GC–MS methodology now explicitly describes the TIC peak-area normalization procedure and states that the resulting values are semi-quantitative. The title and footnote of Table 1 have also been revised to identify the reported percentages as semi-quantitative relative abundances. In addition, relevant statements throughout the Results and Discussion have been modified to avoid implying absolute quantification.

We appreciate the reviewer’s comment, which has improved the accuracy and transparency of the presentation of the GC–MS compositional data.

Yields and compositions. You now report three independent extractions per sample type, but yields (1.1–2.1%) and all compositional percentages are given as single values. Please report both as mean ± SD across the three extractions.

Response: We thank the reviewer for raising this important point. We have clarified the distinction between the independent extraction replicates and the GC–MS analytical measurements. Each EO sample type was obtained from three independent extractions, and the extraction yields are therefore reported as mean ± SD (n = 3). However, due to the limited availability of analytical resources, the GC–MS compositional analysis was performed as a single analytical run for each EO sample. Consequently, analytical replicate variability and SD values cannot be calculated for the GC–MS compositional percentages without additional GC–MS measurements.

We have therefore not generated or reported SD values for the GC–MS composition, as doing so would not be statistically justified. Instead, we have explicitly stated in the Methods and Table 1 footnote that the compositional data represent semi-quantitative relative abundances obtained from TIC peak-area normalization based on a single GC–MS analytical run per sample. We have also clarified that these values are not absolute concentrations and that the lack of analytical replication represents a limitation of the compositional analysis.

We appreciate the reviewer’s comment, which prompted us to clarify the level of replication and the limitations of the GC–MS compositional data.

CLSI standard. M07 is the dilution standard for aerobic bacteria and does not cover filamentous fungi; M38 is the applicable document. Your incubation conditions (25–28 °C, 3–5 days) are appropriate for phytopathogens but constitute an adaptation of the standard, which should be stated as such. Please also cite the current edition with year, specify which medium was used for which organism group (MHB and RPMI are both mentioned without assignment), and give the pH and buffering.

**Response:** We thank the reviewer for this important clarification. We agree that CLSI M07 is applicable to aerobic bacteria, whereas the appropriate CLSI reference for filamentous fungi is M38. We have therefore revised Section 2.5 to cite the current **CLSI M07, 12th edition (2024)** for the bacterial broth microdilution assay and **CLSI M38, 3rd edition (2017)** for the fungal assay.

We have also clarified that the fungal procedure was **adapted from the M38 reference method** to accommodate the growth characteristics of the phytopathogenic fungi investigated in this study, particularly with respect to incubation temperature and duration. The manuscript now explicitly describes these conditions as adaptations rather than as strict application of the clinical CLSI procedure.

In addition, we have specified the culture medium used for each microorganism group and added the corresponding information on medium composition, buffering, and pH in Section 2.5. The revised Methods section now clearly distinguishes the bacterial and fungal assay conditions.

We appreciate the reviewer’s comment, which has improved the methodological clarity and reproducibility of the MIC assay.

Control MIC values. Phenol (3516 µg/mL) and bleach (1875–3750 µg/mL) lie outside the stated dilution series of 100–0.1 µg/mL. Please describe how the controls were diluted and tested. Phenol is also reported as identical (3516 µg/mL) against all eight fungi, which reads as a single fixed endpoint rather than eight independent determinations.

**Response:
We thank the reviewer for pointing out the need to clarify the preparation and testing of the reference controls. We have revised Section 2.5 to distinguish the dilution series used for the essential oils from the separate dilution ranges used for the phenol and sodium hypochlorite controls.**

**The essential oils were tested within the EO dilution series described in the Methods, whereas phenol and sodium hypochlorite were prepared and serially diluted separately at concentrations appropriate for the reference-control experiments. Therefore, their reported MIC values were not restricted to the 0.1–100 µg/mL EO dilution range.**

**We also rechecked the phenol results for all eight fungal isolates. The value of 3516 µg/mL was obtained under the same experimental endpoint conditions for each isolate. We have therefore clarified in the revised manuscript that this value represents the experimentally determined endpoint for phenol under the applied assay conditions, rather than implying that 3516 µg/mL was simply assigned as a fixed value in advance.**

**To avoid ambiguity, the revised Table 4 now identifies phenol and sodium hypochlorite as reference disinfectant controls, rather than conventional antifungal agents. Their results are presented as reference measurements under the experimental conditions and are not used to claim comparative superiority of the essential oils.**

3.2, lines 343–346. The text states that a slight increase in antioxidant activity was observed in the Bethlehem dried sample, but Table 2 gives 70.55 versus 65.02 µg/mL for fresh, which is a decrease in activity. Please correct.

**Response:
We thank the reviewer for identifying this inconsistency. We rechecked the DPPH results and confirmed that the antioxidant activity of the Bethlehem EO decreased after drying, rather than increased. Specifically, the DPPH inhibition decreased from 31.15 ± 0.58% in the fresh EO to 28.21 ± 0.76% in the dried EO at 33.33 µg/mL. Consistently, the corresponding IC₅₀ values, where applicable, indicate lower antioxidant activity for the dried sample because a higher IC₅₀ represents weaker radical-scavenging activity.**

**The erroneous statement describing a slight increase in antioxidant activity in the Bethlehem dried sample has therefore been removed and replaced with a statement describing the observed decrease. The Results and Discussion sections have been revised accordingly.**

Short title. The submission record still carries "ssential Oil Composition…" with the leading E missing. The correction appears not to have been applied in Editorial Manager.

**Response:
We thank the Editor for pointing out this typographical error. The missing initial “E” in the short title has been corrected in the revised manuscript, and the short title now reads “Essential Oil Composition…”. We have also checked the manuscript for consistency. We understand that the short title displayed in Editorial Manager may require correction at the submission-system level, and we would be grateful if the Editorial Office could update it accordingly.**

Section heading. §3.2 reads "Evaluation of the Antioxidant" — please complete ("Antioxidant Activity").

**Response:**

**We thank the Editor for pointing out this incomplete section heading. The heading in Section 3.2 has been corrected from “Evaluation of the Antioxidant” to “Evaluation of the Antioxidant Activity.”**

Graphical abstract placeholder. Lines 20–38 of the main article file contain an empty "Graphical abstract" heading followed by blank lines; the figure is supplied separately. Please remove the placeholder.

**Response: We thank the reviewer for pointing this out. The empty “Graphical abstract” placeholder has been removed from the main manuscript file, while the graphical abstract figure is provided separately as requested.**

Nomenclature and typography. Klebsiella pneumonia → K. pneumoniae (Table 3 and §3.3); missing period after "6.25 µg/mL" at line 54; "two geographically regions" → "two geographically distinct regions" (Abstract, line 58); "rendering to geographical origin" (line 262) → "according to"; "ditribution" → "distribution" (Table 1).

**Response:** **We thank the reviewer for carefully identifying these nomenclature, typographical, and wording issues. All of the indicated corrections have been addressed in the revised manuscript. Specifically, *Klebsiella pneumonia* has been corrected to *K. pneumoniae* in Table 3 and Section 3.3; the missing period after “6.25 µg/mL” has been added; “two geographically regions” has been corrected to “two geographically distinct regions”; “rendering to geographical origin” has been corrected to “according to”; and the misspelling “ditribution” has been corrected to “distribution” in Table 1.**

Nomenclature of the species. Consider noting the currently accepted name Thymbra capitata (L.) Cav. at first mention, since several of your comparator studies use it and the synonymy affects literature retrieval.

**Response:**

We thank the reviewer for this valuable taxonomic suggestion. The currently accepted name, *Thymbra capitata* (L.) Cav., has been noted at the first mention of *Coridothymus capitatus* in the manuscript to clarify the taxonomic synonymy and facilitate comparison with previous studies using the name *Thymbra capitata*.
