# Supplementary material for: Essential oil composition and biological activities of Coridothymus capitatus collected from two locations in Palestine
Source: PLoS One. 2026 Sep 2;21(9):e0357443. doi: 10.1371/journal.pone.0357443 (PMC13537545; doi:10.1371/journal.pone.0357443)
Supplement: Peer review history

# Peer review: Decision Letter 0

**DOI:** 10.1371/journal.pone.0357443.r001

**Authors:** Guadalupe Nevárez-Moorillón

2 Jul 2026

Dear Dr. Bsharat,

Thank you for submitting your manuscript to PLOS ONE. After careful consideration, we feel that it has merit but does not fully meet PLOS ONE’s publication criteria as it currently stands. Therefore, we invite you to submit a revised version of the manuscript that addresses the points raised during the review process.

Please submit your revised manuscript by Aug 16 2026 11:59PM. If you will need more time than this to complete your revisions, please reply to this message or contact the journal office at plosone@plos.org. When you're ready to submit your revision, log on to https://www.editorialmanager.com/pone/ and select the 'Submissions Needing Revision' folder to locate your manuscript file.

- A letter that responds to each point raised by the academic editor and reviewer(s). You should upload this letter as a separate file labeled 'Response to Reviewers'.
- A marked-up copy of your manuscript that highlights changes made to the original version. You should upload this as a separate file labeled 'Revised Manuscript with Track Changes'.
- An unmarked version of your revised paper without tracked changes. You should upload this as a separate file labeled 'Manuscript'.

If you would like to make changes to your financial disclosure, please include your updated statement in your cover letter. Guidelines for resubmitting your figure files are available below the reviewer comments at the end of this letter.

If applicable, we recommend that you deposit your laboratory protocols in protocols.io to enhance the reproducibility of your results. Protocols.io assigns your protocol its own identifier (DOI) so that it can be cited independently in the future. For instructions see: https://journals.plos.org/plosone/s/submission-guidelines#loc-laboratory-protocols. Additionally, PLOS ONE offers an option for publishing peer-reviewed Lab Protocol articles, which describe protocols hosted on protocols.io. Read more information on sharing protocols at https://plos.org/protocols?utm_medium=editorial-email&utm_source=authorletters&utm_campaign=protocols.

As the corresponding author, your ORCID iD is verified in the submission system and will appear in the published article. PLOS supports the use of ORCID, and we encourage all coauthors to register for an ORCID iD and use it as well. Please encourage your coauthors to verify their ORCID iD within the submission system before final acceptance, as unverified ORCID iDs will not appear in the published article. *Only*  the individual author can complete the verification step; PLOS staff *cannot*  verify ORCID iDs on behalf of authors.

We look forward to receiving your revised manuscript.

Kind regards,

Guadalupe Virginia Nevárez-Moorillón, Ph.D.

Academic Editor

PLOS One

Journal requirements:

When submitting your revision, we need you to address these additional requirements.

1. Please ensure that your manuscript meets PLOS ONE's style requirements, including those for file naming. The PLOS ONE style templates can be found at

https://journals.plos.org/plosone/s/file?id=wjVg/PLOSOne_formatting_sample_main_body.pdf and

https://journals.plos.org/plosone/s/file?id=ba62/PLOSOne_formatting_sample_title_authors_affiliations.pdf

If the reviewer comments include a recommendation to cite specific previously published works, please review and evaluate these publications to determine whether they are relevant and should be cited. There is no requirement to cite these works unless the editor has indicated otherwise.

[Note: HTML markup is below. Please do not edit.]

Reviewers' comments:

Reviewer's Responses to Questions

**Comments to the Author**

1. Is the manuscript technically sound, and do the data support the conclusions?

Reviewer #1: Partly

Reviewer #2: Partly

**********

2. Has the statistical analysis been performed appropriately and rigorously?

Reviewer #1: No

Reviewer #2: No

**********

3. Have the authors made all data underlying the findings in their manuscript fully available?

The PLOS Data policy

Reviewer #1: No

Reviewer #2: Yes

**********

4. Is the manuscript presented in an intelligible fashion and written in standard English?

Reviewer #1: No

Reviewer #2: Yes

**********

Reviewer #1: General Evaluation

The manuscript investigates the chemical composition and biological activities of essential oils (EOs) extracted from Coridothymus capitatus collected from two Palestinian regions. The topic is of great interest due to the increasing interest in natural antimicrobial and antioxidant agents. The study combines GC-MS profiling with antioxidant, antibacterial, and antifungal assays, which provides useful preliminary data.

However, the manuscript currently has several methodological, analytical, and presentation weaknesses that should be addressed before publication. Major revisions are recommended.

Major Comments

Introduction

Novelty and Scientific Contribution Need Better Clarification

The manuscript largely confirms already established properties of C. capitatus EO (high thymol/carvacrol content and antimicrobial activity). The authors should better explain, what is genuinely novel in this study, why the Palestinian plant populations are scientifically important, and how this work advances beyond previous Mediterranean chemotype studies. The Introduction should explicitly define the research gap. Currently, the rationale remains descriptive rather than hypothesis-driven.

Materials & methods

Lines 133-135 The description of the plant material is unclear. The authors refer here to “fresh” and “dried” leaves; however, the manuscript does not clearly explain earlier in the Methods section that two different leaf conditions/treatments were included in the experimental design. This should be explicitly clarified before presenting the extraction yields in order to avoid confusion for the reader.

Line 182 The use of phenol and bleach as positive controls for antifungal activity is unusual and insufficiently justified. Standard antifungal agents (e.g., amphotericin B, nystatin, fluconazole, or commercial fungicides) would be more appropriate for comparison and would improve the scientific validity and comparability of the results. The authors should justify their choice of controls or reconsider the experimental design.

Line 185-187 The determination of MIC is insufficiently described. The authors state that fungal growth inhibition was evaluated “visually or by quantifying turbidity/optical density,” but it is unclear which method was ultimately used for each experiment. The methodology should be standardized and described more precisely, including the wavelength used for OD measurements, cutoff criteria for inhibition, and whether measurements were performed spectrophotometrically or by visual inspection alone.

Statistical analysis

The statistical section is insufficient. The authors don’t mention which type of ANOVA, no post hoc test is reported, no p-values are shown, how many were the replicates? The manuscript requires substantially improved statistical treatment.

Results & Discussion

GC-MS Identification Requires More Rigor

Lines 196-207 The GC-MS section currently lacks critical analytical details required for reproducibility and validation. The authors must clarify whether authentic standards were used for compound verification. Additionally, please specify the exact version of the mass spectral library used and the minimum similarity threshold accepted for tentative identification. It is also unclear whether Kovats retention indices were experimentally validated; this must be explicitly stated.

Regarding the data presentation, there are several major flaws in Table 1. Listing numerous compounds as “ND” without proper identification is insufficient; unknown peaks must either be identified, discussed if they are biologically relevant, or omitted entirely if they fall below the significance threshold. Finally, placeholder notation such as “??” is unacceptable in a finalized manuscript and must be corrected.

Minor Comments

Inconsistency in Geographic Locations

There is inconsistency regarding collection sites, most sections refer to Hebron and Bethlehem, but Tables 3 and 4 mention “Ramallah.” Please correct throughout the manuscript.

Table Presentation

Table 1 is overcrowded and difficult to read. Consider, separating major/minor constituents, grouping by terpene class, or moving full chromatographic details to supplementary material.

Table 3 and table 4 lacks SD

References

Finally, the reference list appears outdated. The authors should revise and update their citations to include recent relevant literature, ensuring the discussion aligns with current developments.

Reviewer #2: Methodological concern — statistical analysis and replication

The Methods section (2.6) states that data are expressed as mean ± SEM or mean ± SD, and that ANOVA was used to assess differences. However, the number of biological and technical replicates is never specified for any assay. It is impossible to evaluate the validity of the reported means or the reliability of statistical comparisons without this information. The authors must clarify: (a) how many independent extractions were performed per sample type; (b) how many replicate wells/measurements were used per assay; and (c) whether the ANOVA results (including post-hoc tests and p-values) can be provided in tables or supplementary material. As currently written, Table 2 presents IC₅₀ ± values that appear to be SEM but lack statistical group assignments, and Tables 3–4 report single MIC values with no measure of variability or indication that assays were repeated.

Figure 1 — critical deficiencies

The dose–response figure for the DPPH assay has several serious problems. First, the x-axis is labeled "Conc" without units, which is unacceptable. Second, the concentration range shown (up to ~33 µg/mL) does not reach the reported IC₅₀ values for the EOs (61–70 µg/mL), meaning the curves for the four EO samples never approach 50% inhibition within the plotted range. This makes IC₅₀ determination from the figure impossible to verify and raises doubts about whether the values in Table 2 were obtained by interpolation, extrapolation, or curve fitting. Third, the figure title refers to "Zohif," a term that does not appear anywhere else in the manuscript and appears to be an artifact of a different experiment or a translation error. Fourth, axis labels and legend font sizes are too small. The figure must be entirely rebuilt with appropriate axes, units, full concentration range (at least up to 100 µg/mL as stated in the methods), and a corrected title.

Inconsistency in Table 3 and 4 captions vs. collection sites

Both Table 3 and Table 4 captions state that the EOs were "collected from Ramallah in Palestine." This is incorrect — the study explicitly states material was collected from Hebron and Bethlehem. This is not a minor typo; it suggests that tables may have been recycled or copied from a different dataset, which undermines confidence in the data integrity. The authors must correct this and confirm that the data in both tables correspond to the Hebron and Bethlehem samples described throughout the manuscript.

Comparison with positive controls in antifungal assays (Table 4)

The authors state that the EOs showed "much superior antifungal efficacy" compared to phenol and 3% bleach. However, phenol and bleach are biocidal disinfectants, not standard clinical or agricultural antifungal agents. Their use as positive controls is scientifically inappropriate for an antifungal MIC assay; recognized positive controls such as fluconazole, amphotericin B, or imazalil are expected. Furthermore, the MIC values reported for phenol (3,516 µg/mL) and bleach (1,875 µg/mL) are dramatically higher than those for the EOs, which is not a meaningful comparison and could mislead readers. The authors should either replace these controls with appropriate antifungal agents or provide a clear pharmacological justification for the choice and refrain from drawing superiority conclusions from this comparison.

Antifungal MIC — missing data and unresolved "no effect" results

For Paecilomyces niveus, two EO samples (Hebron fresh and Hebron dry) showed "no effect" at the tested concentrations, yet no upper limit or ">100 µg/mL" designation is provided. The authors should report this as a defined MIC (e.g., >100 µg/mL) and discuss the biological significance of this resistance pattern, given that P. niveus is a known mycotoxin producer. The incomplete inhibition for specific samples also warrants further discussion beyond the single sentence currently offered.

References [5] and [6] in the Introduction cite studies on Juniperus essential oils (Baser et al., 1994; Kokkini et al., 2004), yet they are cited in the context of C. capitatus biology and traditional medicine uses. This is an apparent error — either the wrong references were cited or the citation numbering was shifted during manuscript preparation. All references must be verified for accuracy and relevance.

EO yields are reported (1.1–2.1%) but moisture content of the plant material was not assessed, as acknowledged by the authors. This omission weakens the interpretation of yield differences between fresh and dried samples, since yield on a fresh-weight basis is inherently confounded by water content. At minimum, the authors should provide the approximate moisture content estimated from the weight difference before and after drying, or acknowledge this as a quantitative limitation in both the Methods and Discussion.

Minor Comments

The running title on the manuscript cover page reads "ssential Oil Composition and Biological Activities of Coridothymus capitatus" — the leading "E" is missing. This must be corrected.

Throughout the manuscript, "Bethlehem" is occasionally misspelled as "Beitlehem" (e.g., Table 1 footnote). All place names should be checked and standardized.

Section 3.3 (Antibacterial Activity) contains a near-verbatim repetition of two consecutive paragraphs (lines 313–320 and 321–329), describing essentially the same results for B. cereus and S. aureus twice. One of these paragraphs should be removed.

The term "subjectively" in 2.3 ("identified both subjectively and quantitatively") is likely intended to mean "qualitatively." Please correct.

The DMSO solvent control is mentioned in 2.5 (final concentration 5%) but no results for this control appear in the results section. A statement confirming the absence of inhibitory effects at this DMSO concentration should be included.

For the antibacterial MIC assay, only broth microdilution method reference [26] is cited; however, reference [26] (Hammer et al., 2003) describes antifungal methods for tea tree oil components, not a general antibacterial broth microdilution standard. The appropriate CLSI or EUCAST guideline should be cited instead.

The Conclusion mentions that "drying affected both chemical composition and bioactivity, typically augmenting antibacterial potency." This is an overgeneralization — the antifungal data show a mixed picture (e.g., Hebron fresh outperforms Hebron dry against Botrytis californica). The conclusion should more accurately reflect the nuanced results.

The Declarations section lists "Finding" instead of "Funding." Please correct.

**********

what does this mean?). If published, this will include your full peer review and any attached files.

If you choose “no”, your identity will remain anonymous but your review may still be made public.

**Do you want your identity to be public for this peer review?**  For information about this choice, including consent withdrawal, please see our Privacy Policy

Reviewer #1: No

Reviewer #2: **Yes:** Raul Avila-Sosa

**********

[NOTE: If reviewer comments were submitted as an attachment file, they will be attached to this email and accessible via the submission site. Please log into your account, locate the manuscript record, and check for the action link "View Attachments". If this link does not appear, there are no attachment files.]

To ensure your figures meet our technical requirements, please review our figure guidelines: https://journals.plos.org/plosone/s/figures

You may also use PLOS’s free figure tool, NAAS, to help you prepare publication quality figures: https://journals.plos.org/plosone/s/figures#loc-tools-for-figure-preparation.

NAAS will assess whether your figures meet our technical requirements by comparing each figure against our figure specifications.

# Author comment: Author response to Decision Letter 1

**DOI:** 10.1371/journal.pone.0357443.r002

28 Jul 2026

PONE-D-26-11174

Essential Oil Composition and Biological Activities of Coridothymus capitatus Collected from Two Locations in Palestine

PLOS One

Dear Dr. Bsharat,

Thank you for submitting your manuscript to PLOS ONE. After careful consideration, we feel that it has merit but does not fully meet PLOS ONE’s publication criteria as it currently stands. Therefore, we invite you to submit a revised version of the manuscript that addresses the points raised during the review process.

Please consider the suggestions of both reviewers

Response: We sincerely thank the Editor and the reviewers for their valuable comments and constructive suggestions. We have carefully addressed all comments and revised the manuscript accordingly. A detailed, point-by-point response is provided below, and all changes have been incorporated into the revised manuscript.

We appreciate your time and consideration and hope that the revised manuscript is now suitable for publication in PLOS ONE.

Reviewer #1: General Evaluation

The manuscript investigates the chemical composition and biological activities of essential oils (EOs) extracted from Coridothymus capitatus collected from two Palestinian regions. The topic is of great interest due to the increasing interest in natural antimicrobial and antioxidant agents. The study combines GC-MS profiling with antioxidant, antibacterial, and antifungal assays, which provides useful preliminary data.

However, the manuscript currently has several methodological, analytical, and presentation weaknesses that should be addressed before publication. Major revisions are recommended.

Major Comments

Introduction

Novelty and Scientific Contribution Need Better Clarification

The manuscript largely confirms already established properties of C. capitatus EO (high thymol/carvacrol content and antimicrobial activity). The authors should better explain, what is genuinely novel in this study, why the Palestinian plant populations are scientifically important, and how this work advances beyond previous Mediterranean chemotype studies. The Introduction should explicitly define the research gap. Currently, the rationale remains descriptive rather than hypothesis-driven.

Response: We thank the reviewer for this insightful comment. We agree that the study's originality and scientific importance should be better communicated. The introduction has been considerably revised to clarify the research gap, study hypothesis, and scientific value of studying Palestinian Coridothymus capitatus communities.

We note that previous research has focused on C. capitatus populations from multiple Mediterranean states, using diverse sample sites, phenological phases, extraction procedures, and biological assays, making direct comparisons difficult. Geography has been shown to affect C. capitatus essential oils, but no study has examined the synergistic effects of geographical origin and post-harvest drying on the chemical composition and biological activities of essential oils from different Palestinian populations under uniform experimental conditions.

The Introduction now states that the Hebron and Bethlehem regions' different environmental conditions and post-harvest drying affect volatile secondary metabolite biosynthesis, resulting in significant differences in essential oil composition and biological activities. Instead of verifying C. capitatus essential oil's antioxidant and antibacterial characteristics, this study presents the first systematic comparative examination of fresh and dried leaves from two geographically distinct Palestinian groups. This study integrates chemical characterization with antioxidant, antibacterial, and antifungal property assessments in a single experimental framework to reduce methodological variability and directly evaluate the effects of geographical origin and drying. These findings help us understand Palestinian C. capitatus chemotype diversity and Mediterranean populations of this therapeutic species.

The introduction's final paragraph includes the changes.

Materials & methods

Lines 133-135 The description of the plant material is unclear. The authors refer here to “fresh” and “dried” leaves; however, the manuscript does not clearly explain earlier in the Methods section that two different leaf conditions/treatments were included in the experimental design. This should be explicitly clarified before presenting the extraction yields in order to avoid confusion for the reader.

Response: Thank you for this valuable comment. We have revised the Materials and Methods section to clarify the experimental design. Specifically, we now explicitly state in Section 2.1 that the collected leaves from each location were divided into two portions: one portion was used immediately for essential oil extraction as fresh material, while the other portion was shade-dried and subsequently used for extraction. This clarification has been incorporated before the extraction procedure to avoid confusion.

Line 182 The use of phenol and bleach as positive controls for antifungal activity is unusual and insufficiently justified. Standard antifungal agents (e.g., amphotericin B, nystatin, fluconazole, or commercial fungicides) would be more appropriate for comparison and would improve the scientific validity and comparability of the results. The authors should justify their choice of controls or reconsider the experimental design.

Response: We thank the reviewer for highlighting this important point. We agree that amphotericin B, nystatin, fluconazole or an established commercial fungicide would be more conventional reference chemicals and allow comparison with other antifungal investigations.

Phenol and sodium hypochlorite were selected as widely accessible disinfectants and are utilized in sanitation techniques for the disinfection of tools, equipment and contaminated materials to prevent microbial contamination. They were included to provide a locally relevant and practical benchmark for comparison with the tested plant extracts rather than to represent standard clinical antifungal agents.

The fungal isolates of this study are phytopathogenic fungi isolated and identified from diseased Palestinian plants. The clinical antifungal drugs (e.g., amphotericin B, nystatin and fluconazole) are mainly used for the medically important fungi, not to be used routinely as reference compounds for evaluation of plant pathogenic fungi. Furthermore, a lot of investigations on plant extracts have used phenol and/or sodium hypochlorite as broad-spectrum antibacterial reference disinfectants and not as typical antifungal medications. Therefore, we have edited the text to state that phenol and sodium hypochlorite were used as reference disinfectant controls rather than standard antifungal controls and have stated this as a limitation of the current work.

[Rogawansamy S, Gaskin S, Taylor M, Pisaniello D. An evaluation of antifungal agents for the treatment of fungal contamination in indoor air environments. Int J Environ Res Public Health. 2015 Jun 2;12(6):6319-32. doi: 10.3390/ijerph120606319. PMID: 26042369; PMCID: PMC4483703.]

Copes WE, Ojiambo PS. 2021. Efficacy of Hypochlorite as a Disinfestant Against Fungal Pathogens in Agricultural and Horticultural Plant Production: A Systematic Review and Meta-Analysis. Phytopathology 111(8):1369–1379. https://doi.org/10.1094/PHYTO-05-20-0201-R.

Copes WE, Ojiambo PS. 2021. Efficacy of Hypochlorite in Disinfesting Nonfungal Plant Pathogens in Agricultural and Horticultural Plant Production: A Meta-Analysis. Plant Disease 105:4084–4094. https://doi.org/10.1094/PDIS-09-20-2046-RE.

Gupta AK, Ahmad I, Summerbell RC. 2002. Fungicidal activities of commonly used disinfectants and antifungal pharmaceutical spray preparations against clinical strains of Aspergillus and Candida species. Medical Mycology 40:201–208.

Line 185-187 The determination of MIC is insufficiently described. The authors state that fungal growth inhibition was evaluated “visually or by quantifying turbidity/optical density,” but it is unclear which method was ultimately used for each experiment. The methodology should be standardized and described more precisely, including the wavelength used for OD measurements, cutoff criteria for inhibition, and whether measurements were performed spectrophotometrically or by visual inspection alone.

Response: The methodology was modified after preliminary observations showed that measuring optical density at 600 nm (OD600) was not a reliable indicator of microbial growth in the presence of the oil treatments. The oils caused significant optical interference, producing background artifacts that affected absorbance readings and prevented accurate differentiation between microbial growth and the intrinsic turbidity of the oil. Consequently, OD600 measurements could not be used as a dependable method for assessing growth inhibition. Instead, each well was examined individually using an ocular dissecting microscope to directly assess the presence or absence of visible fungal mycelial growth and bacterial turbidity. This direct visual evaluation provided a more accurate and reproducible assessment of microbial growth, eliminating the interference caused by the oils and allowing for reliable determination of antimicrobial activity.

Statistical analysis

The statistical section is insufficient. The authors don’t mention which type of ANOVA, no post hoc test is reported, no p-values are shown, how many were the replicates? The manuscript requires substantially improved statistical treatment.

Response: All experiments were performed in triplicate (n = 3), and the results are presented as the mean ± standard deviation (SD). IC₅₀ values were calculated by plotting the percentage of DPPH radical scavenging activity against sample concentration using Microsoft Excel. Statistical analyses were performed using one-way analysis of variance (one-way ANOVA), followed by Tukey's multiple comparison post hoc test to determine significant differences among the means. Differences were considered statistically significant at p < 0.05. This has been added and clarified in section 2.6. For Minimum inhibitory concentration (MIC) values were determined as discrete two-fold serial dilution endpoints (e.g., 25, 50, or 100 µg/mL) rather than continuous quantitative measurements. Consequently, conventional inferential statistical analyses are generally not appropriate for comparing MIC values among treatments. To ensure the reliability and reproducibility of the results, all experiments were performed independently in triplicate, and identical MIC values were obtained in all replicates. Therefore, the MIC values are reported directly without statistical comparison, which is consistent with standard practice in antimicrobial susceptibility testing and MIC determination studies

Results & Discussion

GC-MS Identification Requires More Rigor

Lines 196-207 The GC-MS section currently lacks critical analytical details required for reproducibility and validation. The authors must clarify whether authentic standards were used for compound verification. Additionally, please specify the exact version of the mass spectral library used and the minimum similarity threshold accepted for tentative identification. It is also unclear whether Kovats retention indices were experimentally validated; this must be explicitly stated.

Response: We thank the reviewer for this valuable comment. To improve the reproducibility and transparency of the GC–MS analysis, we have revised the Materials and Methods section (section 2.3) by providing additional details regarding compound identification. The EO constituents were identified using a combination of authentic reference standards, mass spectral library matching, and retention index comparison. 15 authentic reference compounds, including the major constituents of Coridothymus capitatus essential oil, were analyzed under identical chromatographic conditions to verify compound identities by comparing both their retention times and mass spectra with those of the sample constituents. The remaining compounds were tentatively identified by comparison of their mass spectra with those contained in the NIST/EPA/NIH Mass Spectral Library (NIST 17) and by comparison of their experimentally determined Kovats retention indices with published literature values obtained on the same stationary phase. A minimum spectral similarity score of 90% was used as the criterion for tentative identification, while compounds with lower similarity scores were further evaluated based on their fragmentation patterns and retention behavior before assignment. The Kovats retention indices were experimentally determined using a homologous series of n-alkanes analyzed under the same chromatographic conditions as the essential oil samples and were compared with published retention index data to further confirm compound identities.

Additionally, we use published retention index data reported by Adams (2007); Adams RP. Identification of Essential Oil Components by Gas Chromatography/Mass Spectrometry. 4.1 ed. Carol Stream, IL, USA: Allured Publishing Corporation; 2007.

Regarding the data presentation, there are several major flaws in Table 1. Listing numerous compounds as “ND” without proper identification is insufficient; unknown peaks must either be identified, discussed if they are biologically relevant, or omitted entirely if they fall below the significance threshold. Finally, placeholder notation such as “??” is unacceptable in a finalized manuscript and must be corrected.

Response: We thank the reviewer for this helpful suggestion. To improve the clarity and readability of the manuscript, the original table has been replaced with a new table summarizing the identified compounds according to their chemical classes (e.g., monoterpene hydrocarbons, oxygenated monoterpenes, sesquiterpene hydrocarbons, oxygenated sesquiterpenes, and phenylpropanoids) In addition, compounds that were not identified or occurred only in trace amounts (<0.1%) have been omitted from the table, except for a few compounds present above this threshold that were retained for completeness as suggested. This presentation provides a clearer overview of the essential oil composition and facilitates comparison of the major chemical classes among the studied samples.

Minor Comments

Inconsistency in Geographic Locations

There is inconsistency regarding collection sites, most sections refer to Hebron and Bethlehem, but Tables 3 and 4 mention “Ramallah.” Please correct throughout the manuscript.

Response: Thank you for pointing out this inconsistency. We have carefully reviewed the manuscript and corrected the geographic locations throughout. The incorrect mention of "Ramallah" in Tables 3 and 4 has been replaced with the correct collection sites, "Hebron" and "Bethlehem," ensuring consistency across the entire manuscript.

Table Presentation

Table 1 is overcrowded and difficult to read. Consider, separating major/minor constituents, grouping by terpene class, or moving full chromatographic details to supplementary material.

Response: We thank the reviewer for this valuable suggestion. To improve the readability of Table 1, we have revised its presentation by grouping the identified constituents according to their terpene classes (monoterpene hydrocarbons, oxygenated monoterpenes, sesquiterpene hydrocarbons, oxygenated sesquiterpenes, and others). In addition, compounds that were not identified or occurred only in trace amounts (<0.1%) have been omitted from the table, except for a few compounds present above this threshold that were retained for completeness. As a result, the revised table is substantially less crowded while preserving all information necessary for interpretation of the chemical composition. We believe that, in its revised form, Table 1 is sufficiently concise and therefore have retained it in the main manuscript rather than moving it to the Supplementary Material.

Question: Table 3 and table 4 lacks SD

Response: Concerning Table 3 and 4:

Minimum inhibitory concentration (MIC) values were determined as discrete two-fold serial dilution endpoints (e.g., 25, 50, or 100 µg/mL) rather than continuous quantitative measurements. Conseq

Attachment Submitted filename: Response Letter 25.7-RA.docx

# Peer review: Decision Letter 1

**DOI:** 10.1371/journal.pone.0357443.r003

**Authors:** Guadalupe Nevárez-Moorillón

12 Aug 2026

Dear Dr. Bsharat,

**Please consider the suggestions done by the reviewer.**

Please submit your revised manuscript by Sep 26 2026 11:59PM. If you will need more time than this to complete your revisions, please reply to this message or contact the journal office at plosone@plos.org. When you're ready to submit your revision, log on to https://www.editorialmanager.com/pone/ and select the 'Submissions Needing Revision' folder to locate your manuscript file.

- A letter that responds to each point raised by the academic editor and reviewer(s). You should upload this letter as a separate file labeled 'Response to Reviewers'.
- A marked-up copy of your manuscript that highlights changes made to the original version. You should upload this as a separate file labeled 'Revised Manuscript with Track Changes'.
- An unmarked version of your revised paper without tracked changes. You should upload this as a separate file labeled 'Manuscript'.

If you would like to make changes to your financial disclosure, please include your updated statement in your cover letter. Guidelines for resubmitting your figure files are available below the reviewer comments at the end of this letter.

If applicable, we recommend that you deposit your laboratory protocols in protocols.io to enhance the reproducibility of your results. Protocols.io assigns your protocol its own identifier (DOI) so that it can be cited independently in the future. For instructions see: https://journals.plos.org/plosone/s/submission-guidelines#loc-laboratory-protocols. Additionally, PLOS ONE offers an option for publishing peer-reviewed Lab Protocol articles, which describe protocols hosted on protocols.io. Read more information on sharing protocols at https://plos.org/protocols?utm_medium=editorial-email&utm_source=authorletters&utm_campaign=protocols.

As the corresponding author, your ORCID iD is verified in the submission system and will appear in the published article. PLOS supports the use of ORCID, and we encourage all coauthors to register for an ORCID iD and use it as well. Please encourage your coauthors to verify their ORCID iD within the submission system before final acceptance, as unverified ORCID iDs will not appear in the published article. *Only*  the individual author can complete the verification step; PLOS staff *cannot*  verify ORCID iDs on behalf of authors.

We look forward to receiving your revised manuscript.

Kind regards,

Guadalupe Virginia Nevárez-Moorillón, Ph.D.

Academic Editor

PLOS One

**Journal Requirements:**

If the reviewer comments include a recommendation to cite specific previously published works, please review and evaluate these publications to determine whether they are relevant and should be cited. There is no requirement to cite these works unless the editor has indicated otherwise.

[Note: HTML markup is below. Please do not edit.]

Reviewers' comments:

Reviewer's Responses to Questions

**Comments to the Author**

Reviewer #1: All comments have been addressed

Reviewer #2: (No Response)

**********

2. Is the manuscript technically sound, and do the data support the conclusions?

Reviewer #1: Yes

Reviewer #2: Partly

**********

3. Has the statistical analysis been performed appropriately and rigorously?

Reviewer #1: Yes

Reviewer #2: No

**********

4. Have the authors made all data underlying the findings in their manuscript fully available?

The PLOS Data policy

Reviewer #1: Yes

Reviewer #2: No

**********

5. Is the manuscript presented in an intelligible fashion and written in standard English?

Reviewer #1: Yes

Reviewer #2: Yes

**********

**Reviewer #1:** The authors have addressed all comments and the manuscript has been significantly improved. Thus, I think it is suitable for publication.

**Reviewer #2:** Thank you for a detailed and courteous response. Several points have been resolved well — the restructuring of Table 1, the correction of the collection sites, the removal of the duplicated antibacterial paragraph, the replacement of the MIC methodology reference, and the more measured Conclusion regarding post-harvest drying. The comments below concern issues that remain open, together with some that emerged from the revision itself.

Major comments

IC₅₀ determination (Table 2, Fig. 1, Abstract, §3.2).

In your response you state that the Table 2 values were estimated by extrapolation. The assay spans 0.25–33 µg/mL, where the oils reach approximately 28–34% inhibition, while the reported IC₅₀ values are 61.16–70.55 µg/mL. Values obtained by projecting roughly twofold beyond the highest measured concentration cannot be reported to two decimal places with error terms, nor carried into the Abstract as findings, because the shape of the curve in the region of 50% inhibition has not been observed. Two routes are available:

(a) Preferred: repeat the DPPH assay over a range that reaches at least 100 µg/mL, so that 50% inhibition falls within the measured data, and report interpolated IC₅₀ values with the fitted model and R² stated.

(b) Acceptable alternative: withdraw the IC₅₀ values entirely and report percentage inhibition at the highest concentration tested (33 µg/mL) for each sample, with SD. This is a legitimate way to present the data you have, and the compositional comparison between chemotypes can still be made on that basis. The Abstract, §3.2, and the Conclusion would need to be revised accordingly, including the comparisons with the Moroccan, Libyan, and Tunisian studies, which are IC₅₀-based and would no longer be commensurable.

Please also state explicitly in §2.4 the concentration range actually assayed and the method used to derive IC₅₀ (linear regression, four-parameter fit, or other).

Reported standard deviations (Table 2).

Each of the five SDs is exactly 5.00% of its mean (61.16 ± 3.1; 65.02 ± 3.25; 69.26 ± 3.46; 70.55 ± 3.52; 4.73 ± 0.23). Independent triplicate measurements do not yield a constant coefficient of variation across samples. Please recalculate the dispersion from the raw absorbance data and provide those data — all replicates, all concentrations, all samples — as supplementary material, consistent with your Data Availability statement.

Discrepancy between Figure 1 and Table 2.

The Bethlehem-fresh curve lies above the Hebron-fresh curve at every plotted concentration (approximately 34.5% versus 31% at 33 µg/mL), yet Table 2 reports Hebron fresh as the most active sample. Since §3.2 attributes the higher activity of Hebron fresh to its elevated thymol content, the interpretation depends on which source is correct. Please confirm the ranking and revise whichever presentation is in error. The Trolox curve is a separate concern: reaching approximately 81% inhibition at 3.3 µg/mL, it implies an IC₅₀ near 2 µg/mL rather than the tabulated 4.73 µg/mL.

Magnitude of the MIC values.

Antibacterial MICs of 25–100 µg/mL and antifungal MICs as low as 6.25 µg/mL are one to three orders of magnitude below the range generally reported for thymol- and carvacrol-rich Lamiaceae essential oils, which typically falls between 0.25 and 5 mg/mL. Your DPPH results are displaced in the same direction relative to the comparator studies you cite. Please verify the calculation of final in-well concentrations and confirm that the 100 µL of inoculum added to each 100 µL of diluted oil has been accounted for in the reported values — this alone represents a twofold factor. Please also confirm the units (µg/mL versus µL/mL) and state how the oil mass was determined for the stock solution.

Undertakings not carried into the manuscript.

Four items promised in the response letter are absent from the revised text:

The superiority claim persists at lines 443–444, where the EOs are said to show "much greater antifungal efficacy at slightly reduced dosages." A difference of 6.25 versus 3516 µg/mL is roughly 560-fold, not slight, and the comparison is against controls you have now agreed are not antifungal agents. Please remove the comparative claim and simply report both sets of values.

The stated limitation regarding phenol and bleach as reference disinfectants does not appear in the text. Please add it to §2.5 and to the Discussion.

Table 4 still reports "no effect" for P. niveus. Please replace with ">100 µg/mL" in the table itself.

No statistical group assignments, p-values, or post-hoc results appear. Having described one-way ANOVA with Tukey's test in §2.6, please add superscript letters to Table 2 denoting significantly different groups, report the relevant p-values, and provide the full ANOVA output as supplementary material.

Reference accuracy.

The response states that all citations were verified. The following remain incorrect:

[5] and [16] are the same publication (Saoulajan et al., Trends Food Sci Technol 2022;129:463–491), cited twice under different numbers.

[14] (Roberts et al., bioherbicides for weed control) supports a sentence about Holy Land chemotype profiles.

[37] (Tepe et al., Clinopodium extracts) and [38] (Lagouri & Boskou, spice screening) are cited for enzymatic conversion of γ-terpinene and p-cymene into thymol and carvacrol; neither addresses that pathway.

[40] (Bhatt et al., doxycycline pharmacokinetics in Mycoplasma) is cited at line 340 for the antioxidant mechanism of thymol and carvacrol.

[35] — the author list does not correspond to Arch Biochem Biophys 187(2):307–314; please verify against the original.

[27] is undated and cites M07-A10, a superseded edition (see m5 below).

Please carry out a full citation-by-citation check rather than a spot correction.

Minor comments

Table 1 does not balance. The five class subtotals sum to exactly 100.00 in every column, while "Total identified" is given as 99.32–99.68. The difference equals the "Others" row in each case, indicating that "Others" represents the unidentified remainder — yet two identified compounds (nepetalactone, cis-carvyl propanoate) are listed under a heading "Other compounds." Please separate identified minor constituents from the unidentified fraction. Independently, the Hebron-fresh monoterpene hydrocarbons sum to 25.56 rather than the stated 25.68, and the oxygenated monoterpenes to 68.88 rather than 69.03. Please recheck all subtotals.

Nepetalactone. Its presence in a Thymbra/Coridothymus oil is unusual. Please confirm the identification against an authentic standard or a reported RI on the same stationary phase, or reclassify it as tentative.

Semi-quantification. The percentages appear to derive from TIC area normalisation without FID data or response factors. Please state this explicitly and describe the composition as semi-quantitative.

Yields and compositions. You now report three independent extractions per sample type, but yields (1.1–2.1%) and all compositional percentages are given as single values. Please report both as mean ± SD across the three extractions.

CLSI standard. M07 is the dilution standard for aerobic bacteria and does not cover filamentous fungi; M38 is the applicable document. Your incubation conditions (25–28 °C, 3–5 days) are appropriate for phytopathogens but constitute an adaptation of the standard, which should be stated as such. Please also cite the current edition with year, specify which medium was used for which organism group (MHB and RPMI are both mentioned without assignment), and give the pH and buffering.

Control MIC values. Phenol (3516 µg/mL) and bleach (1875–3750 µg/mL) lie outside the stated dilution series of 100–0.1 µg/mL. Please describe how the controls were diluted and tested. Phenol is also reported as identical (3516 µg/mL) against all eight fungi, which reads as a single fixed endpoint rather than eight independent determinations.

3.2, lines 343–346. The text states that a slight increase in antioxidant activity was observed in the Bethlehem dried sample, but Table 2 gives 70.55 versus 65.02 µg/mL for fresh, which is a decrease in activity. Please correct.

Short title. The submission record still carries "ssential Oil Composition…" with the leading E missing. The correction appears not to have been applied in Editorial Manager.

Section heading. §3.2 reads "Evaluation of the Antioxidant" — please complete ("Antioxidant Activity").

Graphical abstract placeholder. Lines 20–38 of the main article file contain an empty "Graphical abstract" heading followed by blank lines; the figure is supplied separately. Please remove the placeholder.

Nomenclature and typography. Klebsiella pneumonia → K. pneumoniae (Table 3 and §3.3); missing period after "6.25 µg/mL" at line 54; "two geographically regions" → "two geographically distinct regions" (Abstract, line 58); "rendering to geographical origin" (line 262) → "according to"; "ditribution" → "distribution" (Table 1).

Nomenclature of the species. Consider noting the currently accepted name Thymbra capitata (L.) Cav. at first mention, since several of your comparator studies use it and the synonymy affects literature retrieval.

**********

what does this mean?). If published, this will include your full peer review and any attached files.

If you choose “no”, your identity will remain anonymous but your review may still be made public.

**Do you want your identity to be public for this peer review?**  For information about this choice, including consent withdrawal, please see our Privacy Policy

Reviewer #1: No

Reviewer #2: No

**********

[NOTE: If reviewer comments were submitted as an attachment file, they will be attached to this email and accessible via the submission site. Please log into your account, locate the manuscript record, and check for the action link "View Attachments". If this link does not appear, there are no attachment files.]

To ensure your figures meet our technical requirements, please review our figure guidelines: https://journals.plos.org/plosone/s/figures

You may also use PLOS’s free figure tool, NAAS, to help you prepare publication quality figures: https://journals.plos.org/plosone/s/figures#loc-tools-for-figure-preparation.

NAAS will assess whether your figures meet our technical requirements by comparing each figure against our figure specifications.

# Author comment: Author response to Decision Letter 2

**DOI:** 10.1371/journal.pone.0357443.r004

17 Aug 2026

PONE-D-26-11174R1

Essential Oil Composition and Biological Activities of Coridothymus capitatus Collected from Two Locations in Palestine

PLOS One

Dear Dr. Bsharat,

Thank you for submitting your manuscript to PLOS ONE. After careful consideration, we feel that it has merit but does not fully meet PLOS ONE’s publication criteria as it currently stands. Therefore, we invite you to submit a revised version of the manuscript that addresses the points raised during the review process.

Please consider the suggestions done by the reviewer.

Response: Thank you for the reviewer’s suggestions. We have carefully considered and addressed all of the comments raised by the reviewer and revised the manuscript accordingly. All relevant changes have been incorporated into the revised manuscript.

Reviewer #1: The authors have addressed all comments and the manuscript has been significantly improved. Thus, I think it is suitable for publication.

Response: We sincerely thank the reviewer for carefully evaluating our revised manuscript and for the positive assessment. We greatly appreciate the reviewer’s constructive comments and suggestions throughout the review process, which helped us improve the clarity, accuracy, and overall quality of the manuscript. We are grateful for the reviewer’s recommendation that the manuscript is suitable for publication.

Reviewer #2: Thank you for a detailed and courteous response. Several points have been resolved well — the restructuring of Table 1, the correction of the collection sites, the removal of the duplicated antibacterial paragraph, the replacement of the MIC methodology reference, and the more measured Conclusion regarding post-harvest drying. The comments below concern issues that remain open, together with some that emerged from the revision itself.

Major comments

IC₅₀ determination (Table 2, Fig. 1, Abstract, §3.2).

In your response you state that the Table 2 values were estimated by extrapolation. The assay spans 0.25–33 µg/mL, where the oils reach approximately 28–34% inhibition, while the reported IC₅₀ values are 61.16–70.55 µg/mL. Values obtained by projecting roughly twofold beyond the highest measured concentration cannot be reported to two decimal places with error terms, nor carried into the Abstract as findings, because the shape of the curve in the region of 50% inhibition has not been observed. Two routes are available:

(a) Preferred: repeat the DPPH assay over a range that reaches at least 100 µg/mL, so that 50% inhibition falls within the measured data, and report interpolated IC₅₀ values with the fitted model and R² stated.

(b) Acceptable alternative: withdraw the IC₅₀ values entirely and report percentage inhibition at the highest concentration tested (33 µg/mL) for each sample, with SD. This is a legitimate way to present the data you have, and the compositional comparison between chemotypes can still be made on that basis. The Abstract, §3.2, and the Conclusion would need to be revised accordingly, including the comparisons with the Moroccan, Libyan, and Tunisian studies, which are IC₅₀-based and would no longer be commensurable.

Please also state explicitly in §2.4 the concentration range actually assayed and the method used to derive IC₅₀ (linear regression, four-parameter fit, or other).

Response: Thank you for this helpful suggestion. We have revised the manuscript accordingly and have withdrawn the IC₅₀ values from the Abstract, Section 3.2, Table 2, and Conclusion. Instead, DPPH radical-scavenging activity is now reported as the percentage inhibition at the highest experimentally tested concentration (33.33 µg/mL), expressed as mean ± SD (n = 3), for each essential-oil sample.

We have also revised Section 2.4 to explicitly state that the DPPH assay was performed over the concentration range of 0.333–33.33 µg/mL. Because 50% inhibition was not consistently achieved across all essential-oil samples within this experimentally tested range, IC₅₀ values were not calculated or extrapolated. Consequently, no linear regression, four-parameter logistic model, or other IC₅₀-fitting method was used.

In addition, the IC₅₀-based comparisons with previously published Moroccan, Libyan, and Tunisian C. capitatus/T. capitatus essential oils have been removed from Section 3.2, since direct comparison between IC₅₀ values reported in those studies and percentage inhibition measured at 33.33 µg/mL in the present study would not be methodologically commensurable. The Abstract and Conclusion have likewise been revised to reflect this change.

Reported standard deviations (Table 2).

Each of the five SDs is exactly 5.00% of its mean (61.16 ± 3.1; 65.02 ± 3.25; 69.26 ± 3.46; 70.55 ± 3.52; 4.73 ± 0.23). Independent triplicate measurements do not yield a constant coefficient of variation across samples. Please recalculate the dispersion from the raw absorbance data and provide those data — all replicates, all concentrations, all samples — as supplementary material, consistent with your Data Availability statement.

Response: Thank you for identifying this important issue. We agree that the previously reported standard deviations were not appropriately derived from the independent replicate measurements. We have therefore completely recalculated the DPPH radical-scavenging results from the original raw absorbance measurements.

The revised analysis was performed using the individual absorbance values recorded at 517 nm for each replicate at each tested concentration. For each sample and concentration, the mean absorbance and standard deviation were calculated from the three independent replicate measurements. Percentage DPPH inhibition was then calculated for each replicate using the DPPH control absorbance according to the equation:

% inhibition = [(A_control − A_sample)/A_control] × 100.

The mean ± SD values were subsequently calculated from the three replicate inhibition values. Thus, the revised SDs are derived from the actual experimental replicate measurements and are no longer based on a fixed coefficient of variation.

We have also added the complete raw DPPH dataset as Supplementary Material. The supplementary file contains the individual absorbance measurements for all three replicates, all tested concentrations, all four Coridothymus capitatus essential-oil samples (Hebron-fresh, Hebron-dry, Bethlehem-fresh, and Bethlehem-dry), and the Trolox control. The corresponding calculated percentage-inhibition values are also provided to ensure complete transparency and reproducibility of the analysis.

In accordance with our revised analysis, the previously reported IC₅₀ values have been removed from the manuscript. Because 50% inhibition was not consistently achieved by all essential-oil samples within the experimentally tested concentration range, IC₅₀ values were not calculated or extrapolated. No linear regression, four-parameter logistic model, or other curve-fitting procedure was therefore used to derive IC₅₀ values.

Table 2 has been revised accordingly and now reports DPPH inhibition at the highest experimentally tested concentration (33.33 µg/mL) as mean ± SD based on the independent replicate measurements. The Abstract, Section 3.2, and Conclusion have also been revised to remove the previous IC₅₀-based statements.

The complete raw dataset and the corresponding calculations are provided in Supplementary Table S1.

Discrepancy between Figure 1 and Table 2.

The Bethlehem-fresh curve lies above the Hebron-fresh curve at every plotted concentration (approximately 34.5% versus 31% at 33 µg/mL), yet Table 2 reports Hebron fresh as the most active sample. Since §3.2 attributes the higher activity of Hebron fresh to its elevated thymol content, the interpretation depends on which source is correct. Please confirm the ranking and revise whichever presentation is in error. The Trolox curve is a separate concern: reaching approximately 81% inhibition at 3.3 µg/mL, it implies an IC₅₀ near 2 µg/mL rather than the tabulated 4.73 µg/mL.

Response: Thank you for identifying this discrepancy. We carefully rechecked the raw absorbance data and confirmed that the labels for the Bethlehem-fresh and Hebron-fresh samples had inadvertently been interchanged in the previous presentation. This labeling error has now been corrected. Based on the raw absorbance data, Hebron-fresh EO showed the highest DPPH radical-scavenging activity at the highest tested concentration (33.33 µg/mL), followed by Bethlehem-fresh, Hebron-dry, and Bethlehem-dry EOs, with values of 34.40 ± 0.85%, 31.15 ± 0.58%, 29.58 ± 1.00%, and 28.21 ± 0.76%, respectively. Figure 1, Table 2, and the corresponding discussion have been revised to reflect this corrected ranking. The statement linking the higher activity of Hebron-fresh EO to its chemical composition has also been retained only in the context of the corrected data and appropriately qualified.

We also rechecked the Trolox data against the original replicate absorbance measurements. The previously reported Trolox IC₅₀ value of 4.73 µg/mL was not consistent with the raw data and has therefore been removed. The raw data show 40.51 ± 0.91% inhibition at 1.667 µg/mL and 55.98 ± 1.35% inhibition at 2.333 µg/mL, indicating that 50% inhibition was reached between these concentrations. In accordance with the revised approach of avoiding IC₅₀ calculations when they are not necessary for the comparison, we have removed the previously reported Trolox IC₅₀ value rather than introducing a new fitted value. Trolox is now presented as a positive control based on its concentration-dependent DPPH radical-scavenging activity.

The revised manuscript and Supplementary Material now report the raw replicate absorbance measurements and the recalculated percentage inhibition values, allowing the reported results to be independently verified.

Magnitude of the MIC values.

Antibacterial MICs of 25–100 µg/mL and antifungal MICs as low as 6.25 µg/mL are one to three orders of magnitude below the range generally reported for thymol- and carvacrol-rich Lamiaceae essential oils, which typically falls between 0.25 and 5 mg/mL. Your DPPH results are displaced in the same direction relative to the comparator studies you cite. Please verify the calculation of final in-well concentrations and confirm that the 100 µL of inoculum added to each 100 µL of diluted oil has been accounted for in the reported values — this alone represents a twofold factor. Please also confirm the units (µg/mL versus µL/mL) and state how the oil mass was determined for the stock solution.

Response: Thank you for raising this important point. We re-examined the original experimental records and identified an error in the description of the EO stock-solution preparation in the revised manuscript. For each assay, 20 mg of EO was accurately weighed and diluted to a final volume of 100 mL to obtain a 200 µg/mL stock solution. The antibacterial stock solutions were prepared in Mueller–Hinton broth containing 20% (v/v) DMSO, whereas the antifungal stock solutions were prepared in RPMI 1640 medium containing 20% (v/v) DMSO.

Two-fold serial dilutions were prepared in the corresponding assay medium, producing EO concentrations of 100, 50, 25, 12.5, 6.25, 3.125, 1.563, 0.781, 0.391, and 0.195 µg/mL before inoculation. All dilutions were prepared aseptically in sterile 96-well microtiter plates. The maximum DMSO concentration was 10% (v/v) in the first well before inoculation and decreased proportionally with each subsequent two-fold dilution. Corresponding solvent controls containing equivalent DMSO concentrations without EO were included to determine whether DMSO affected microbial growth.

The description and concentration calculations have been corrected in the revised manuscript. We thank the reviewer for identifying this issue, which allowed us to improve the clarity and reproducibility of the antimicrobial assay.

Question: Undertakings not carried into the manuscript.

Four items promised in the response letter are absent from the revised text:

The superiority claim persists at lines 443–444, where the EOs are said to show "much greater antifungal efficacy at slightly reduced dosages." A difference of 6.25 versus 3516 µg/mL is roughly 560-fold, not slight, and the comparison is against controls you have now agreed are not antifungal agents. Please remove the comparative claim and simply report both sets of values.

Response: We acknowledge that it was misleading to call the EO concentrations “slightly reduced” and that direct comparison of efficacy with phenol and bleach was inappropriate since these were included only as practical disinfection reference controls, and not as conventional antifungal agents or agricultural fungicides. We have therefore deleted the statement that the EOs showed ‘much greater antifungal efficacy at slightly reduced dosages’ and all claims of comparative superiority.

The stated limitation regarding phenol and bleach as reference disinfectants does not appear in the text. Please add it to §2.5 and to the Discussion.

Response: Thank you for pointing out this omission. We have now explicitly stated the limitation associated with using phenol and commercial bleach as reference disinfectants in both Section 2.5 and the Discussion. We clarified that these substances are neither conventional antifungal agents nor agricultural fungicides and that their MIC values were included only as descriptive reference values under the conditions of the present assay. Consequently, they were not used to establish comparative superiority or equivalent efficacy of the EOs.

Table 4 still reports "no effect" for P. niveus. Please replace with ">100 µg/mL" in the table itself.

Response: Thank you for pointing this out. We have revised Table 4 accordingly. The entries previously reported as “no effect” for Paecilomyces niveus have been replaced with “>100 µg/mL”, indicating that no inhibition was observed at the highest EO concentration tested (100 µg/mL).

No statistical group assignments, p-values, or post-hoc results appear. Having described one-way ANOVA with Tukey's test in §2.6, please add superscript letters to Table 2 denoting significantly different groups, report the relevant p-values, and provide the full ANOVA output as supplementary material.

Response: Thank you for this helpful comment. We have added statistical group assignments to Table 2 using superscript letters to indicate significant differences among the four essential-oil samples at 33.33 µg/mL. We also report the one-way ANOVA result (F(3,8) = 35.49, p < 0.001) and the corresponding Tukey multiple-comparison results. The complete ANOVA summary and Tukey grouping have been added to the Supplementary Material as Supplementary Table S5. The revised Table 2 and corresponding statistical description in the Results section have been updated accordingly.

Reference accuracy.

The response states that all citations were verified. The following remain incorrect:

[5] and [16] are the same publication (Saoulajan et al., Trends Food Sci Technol 2022;129:463–491), cited twice under different numbers.

Response: Thank you for identifying this error. We confirmed that References [5] and [16] were inadvertently duplicated. We have retained Reference [5] for the general background information on C. capitatus and replaced the duplicated Reference [16] with a more appropriate regional reference addressing the chemotypic variation of C. capitatus in Israel and the West Bank. The corresponding citation in the manuscript and the reference list have been corrected accordingly.

[14] (Roberts et al., bioherbicides for weed control) supports a sentence about Holy Land chemotype profiles.

Response: We thank the reviewer for identifying this citation issue. We agree that the original reference did not directly support the statement concerning the geographical variation in the chemical composition of C. capitatus essential oils. We have therefore replaced the inappropriate reference with El Ouariachi et al. (2011), which specifically investigated the chemical composition of Thymus capitatus essential oil and its biological properties. The corresponding sentence has also been revised to more accurately state that the chemical composition of C. capitatus essential oil can vary geogr

Attachment Submitted filename: Response Letter 14.8-OB.docx

# Editor report: Decision Letter 2

**DOI:** 10.1371/journal.pone.0357443.r005

**Authors:** Guadalupe Nevárez-Moorillón

19 Aug 2026

<p>Essential Oil Composition and Biological Activities of Coridothymus capitatus Collected from Two Locations in Palestine

PONE-D-26-11174R2

Dear Dr. Bsharat,

We’re pleased to inform you that your manuscript has been judged scientifically suitable for publication and will be formally accepted for publication once it meets all outstanding technical requirements.

Within one week, you’ll receive an e-mail detailing the required amendments. When these have been addressed, you’ll receive a formal acceptance letter and your manuscript will be scheduled for publication.

An invoice will be generated when your article is formally accepted. Please note, if your institution has a publishing partnership with PLOS and your article meets the relevant criteria, all or part of your publication costs will be covered. Please make sure your user information is up-to-date by logging into Editorial Manager at Editorial Manager® and clicking the ‘Update My Information' link at the top of the page. For questions related to billing, please contact billing support.

If your institution or institutions have a press office, please notify them about your upcoming paper to help maximize its impact. If they’ll be preparing press materials, please inform our press team as soon as possible -- no later than 48 hours after receiving the formal acceptance. Your manuscript will remain under strict press embargo until 2 pm Eastern Time on the date of publication. For more information, please contact onepress@plos.org.

Kind regards,

Guadalupe Virginia Nevárez-Moorillón, Ph.D.

Academic Editor

PLOS One

Additional Editor Comments (optional):

Reviewers' comments:

# Editor report: Acceptance letter

**DOI:** 10.1371/journal.pone.0357443.r006

**Authors:** Guadalupe Nevárez-Moorillón

PONE-D-26-11174R2

PLOS One

Dear Dr. Bsharat,

I'm pleased to inform you that your manuscript has been deemed suitable for publication in PLOS One. Congratulations! Your manuscript is now being handed over to our production team.

At this stage, our production department will prepare your paper for publication. This includes ensuring the following:

* All references, tables, and figures are properly cited

* All relevant supporting information is included in the manuscript submission,

* There are no issues that prevent the paper from being properly typeset

You will receive further instructions from the production team, including instructions on how to review your proof when it is ready. Please keep in mind that we are working through a large volume of accepted articles, so please give us a few days to review your paper and let you know the next and final steps.

Lastly, if your institution or institutions have a press office, please let them know about your upcoming paper now to help maximize its impact. If they'll be preparing press materials, please inform our press team within the next 48 hours. Your manuscript will remain under strict press embargo until 2 pm Eastern Time on the date of publication. For more information, please contact onepress@plos.org.

You will receive an invoice from PLOS for your publication fee after your manuscript has reached the completed accept phase. If you receive an email requesting payment before acceptance or for any other service, this may be a phishing scheme. Learn how to identify phishing emails and protect your accounts at https://explore.plos.org/phishing.

If we can help with anything else, please email us at customercare@plos.org.

Thank you for submitting your work to PLOS One and supporting open access.

Kind regards,

PLOS One Editorial Office Staff

on behalf of

Dr. Guadalupe Virginia Nevárez-Moorillón

Academic Editor

PLOS One
